# Integrating genome‐wide traits and multi‐loci phylogeny to investigate orchid evolution—A case study on Pleurothallidinae

**DOI:** 10.1111/tpj.70281

**Published:** 2025-06-20

**Authors:** Pavel Trávníček, Jan Ponert, Marcos Vinicius Dantas‐Queiroz, Zuzana Chumová

**Affiliations:** ^1^ Institute of Botany of the Czech Academy of Sciences Zámek 1 Průhonice CZ‐25243 Czech Republic; ^2^ Prague Botanical Garden Trojská 800/196 Prague CZ‐17100 Czech Republic; ^3^ Department of Experimental Plant Biology, Faculty of Science Charles University Viničná 5 Prague CZ‐12844 Czech Republic

**Keywords:** Andes, endoreplication, evolution, GC content, genome size, HybSeq, Pleurothallidinae, spatial analysis

## Abstract

Rapidly radiated groups are usually accompanied by unclear lineage and taxa delineation, which complicates their better understanding in terms of biodiversity, evolutionary processes, and taxonomic treatment. The most species‐rich orchid subtribe, Pleurothallidinae, exemplifies an extremely diverse group with a complex evolutionary history associated with Andean orography. Here we combined multi‐loci phylogeny reconstruction (HybSeq), genome‐wide traits (inferred by flow cytometry), spatial analyses, and biogeography to investigate the evolutionary intricacy of one clade of Pleurothallidinae orchids. To achieve deep insights, we performed multiple species tree reconstruction approaches with the implementation of custom scripts to reveal sources of topological discrepancies and alternative evolutionary scenarios. The phylogeny clearly resolves the delimitation of the main evolutionary lineages corresponding to the accepted genera, with the exception of the genus *Specklinia*, which is divided into three distinct monophyletic lineages whose taxonomic treatment is proposed. Genome‐wide characters (especially genome size) show an association with precipitation seasonality in a geographical context, and partial endoreplication, a unique character of orchids, is geographically restricted to the Andes, Central America, and the Caribbean. Specifically, the Andean region exemplifies the prevalence of bigger genome size and higher GC content, resulting from a higher proportion of species with partial endoreplication. The Andean origin of the clade was also revealed by biogeographic analysis. Our comprehensive approach has provided deep insights into the evolution of this clade and may be a useful tool for unraveling the intricate evolutionary history of similarly complex lineages.

## Significance Statement

The Pleurothallidinae orchids exemplify an exceptionally species‐rich monophyletic group, marked by rapid evolutionary radiation and persistent taxonomic challenges. Through an integrative approach utilizing advanced methods, we provide a comprehensive framework to unravel their evolutionary history and resolve similar complexities in other plant groups, offering valuable insights for future taxonomic and evolutionary research.

## INTRODUCTION

The Orchidaceae (orchids) are one of the three megadiverse families of angiosperms, together with the Asteraceae and Fabaceae, and include more than 31 000 recognized species (POWO, 2023), representing nearly 10% of all flowering plants. The orchid family consists of five subfamilies, with Epidendroideae being the most diverse, accounting for more than half of all orchid species (Pridgeon, 2005). In addition to the two most species‐rich genera *Bulbophyllum* (~2171 recognized species) and *Dendrobium* (~1625 recognized species), the subfamily includes the subtribe Pleurothallidinae, which, with more than 5700 recognized species, is the most species‐rich group of closely related plants and accounts for almost 20% of the species diversity of all orchids. Although the species diversity of the subtribe is roughly estimated, the concept of its genera is a matter of debate. This is due to enormous species richness arising over a relatively short evolutionary time‐span (~20 Ma; Pérez‐Escobar et al., 2017) driven by rapid evolution, accompanied by poor morphological delimitation of taxa. Nevertheless, the classification based on morphology has played a leading role that stands behind the attempts to categorize the taxa, which was crowned by the monographer of the subtribe Pleurothallidinae, Dr. C.A. Luer, the author of the outstandingly detailed *Icones Pleurothallidinarum* (32 parts within Monographs in Systematic Botany, Missouri Botanical Garden; 1986–2012). He and other authors of this age have started with a relatively artificial system of genera based primarily on morphological similarities.

It was only with the introduction of advanced molecular techniques that the first insights were obtained to determine evolutionary relationships and better delimitation of taxa more precisely (starting with the work of Pridgeon & Chase, 2001). Several studies have used classical sequencing approaches (mostly internal transcribed spacer and chloroplast matK sequence data) in combination with morphological data to reconstruct phylogenetic relationships within Pleurothallidinae (reviewed in Karremans, 2016 and Karremans & Vieira‐Uribe, 2020). These studies significantly improved our knowledge of relationships within this group, especially at the level of taxonomic circumscription of genera. However, some lineages commonly lack significant support, and findings from different loci are frequently contradicting. In a short time, it became evident that the deterministic power of classically used molecular markers was exhausted, and only the use of next‐generation sequencing (NGS)‐based techniques, associated with other lines of evidence, could further shed light on the complexity of this subtribe (Bogarín et al., 2018; Chumová et al., 2021).

For instance, our previous studies have shown that evaluating multiple genome‐wide data in a relatively easy way using flow cytometry is useful for grasping evolutionary relationships in orchids (Chumová et al., 2021; Trávníček et al., 2019). In particular, the so‐called partial endoreplication, a unique process of intraindividual polyploidization that occurs exclusively in orchids (Brown et al., 2017; Leitch & Dodsworth, 2017), has consistently been shown, along with genome size, to serve as a distinguishing trait for orchid taxa (e.g., Trávníček et al., 2015, 2019). In contrast, chromosome number has been shown to be a negligible factor modulating genome size variation in Pleurothallidinae orchids (Chumová et al., 2021). In this way, the deterministic power of the NGS‐based phylogeny combined with genome‐wide traits has proven to be an effective tool for unraveling the complex history of the subtribe Pleurothallidinae (Chumová et al., 2021). By constructing the first subtribe‐wide phylogeny using many low‐copy nuclear genes, Chumová et al. (2021) successfully partitioned the subtribe's enormous species richness into several clades that show strong support across multiple gene trees. One of these clades, labeled as clade D, consists of morphologically highly heterogeneous genera, and the above‐mentioned results indicated several discrepancies from the current taxonomic concept. However, Chumová et al. (2021) also concluded that detailed studies devoted to specific clades should help to increase the number of genes involved in the phylogenies and thus better understand the relationships within them. Therefore, this study aims to grasp the evolution and understand the systematics in a complex way within clade D that includes approximately 420 accepted species (POWO, 2023) in about seven genera that have recently been found to be tricky ones with still unresolved taxonomy. We also include clade C, which consists of the single genus *Andinia* Luer (including approximately 75 accepted species; POWO, 2023) and is recognized as a sister clade to clade D (Chumová et al., 2021). Despite our study not being *a priori* targeted to cope with the exceptional clade in terms of species richness, it is pretty much more oriented to inter‐connect various methodological approaches in order to solve the backbone taxonomy from multiple points of view.

### Taxonomic backgrounds

Some genera of these clades were previously recognized mostly based on their conspicuous morphological uniqueness, like *Dryadella* Luer, *Scaphosepalum* Pfitzer in H.G.A. Engler & K.A.E. Prantl, *Plastystele* Schltr., and *Teagueia* (Luer) Luer. Phylogenetic studies based on various DNA sequence data later revealed that *Dryadella* includes also the monospecific genus *Incaea* Luer (Karremans et al., 2016) and that *Rubellia* forms a separate sister clade to *Teagueia* and *Plastystele* (Chumová et al., 2021). Three other genera (*Andinia* (Luer) Luer, *Andreettaea* Luer, *Specklinia* Lindl.) have been recognized relatively recently based mostly on DNA sequence.

*Andinia* Luer was first proposed to accommodate two morphologically distinct species (Luer, 1991, 2000). DNA‐based phylogenetic studies revealed that this group is related to other groups of Pleurothallidinae and resulted in a significant expansion of *Andinia* (Pridgeon & Chase, 2001; Wilson et al., 2017). The monophyly of this genus was later supported by next‐generation sequence data (Chumová et al., 2021). However, due to a significant morphological heterogeneity and infrageneric structure in molecular datasets, the genus *Andinia* was recently proposed to be divided into nine small genera (Szlachetko et al., 2022).

The genus *Andreettaea* Luer was originally created to accommodate a single species, *A. ocellus*, which has unique flower morphology (Luer et al.,^1978^; nevertheless, recent sequencing of nuclear ribosomal internal transcribed spacer and chloroplast matK placed *A. ocellus* within *Muscarella* (Doucette et al., 2022), so both genera were taxonomically merged under *Andreettaea*, which has priority (Doucette, 2022).

The genus *Specklinia* was originally proposed by Lindley (Lindley, 1830); nevertheless, it was reduced in a synonymy with *Pleurothallis* (e.g. Luer, 1986) until the DNA‐based studies resulted in the reappraisal of this group at generic level (Karremans et al., 2016; Luer, 2006; Pridgeon & Chase, 2001) which now accommodates several morphologically unique groups which were previously sometimes treated as separate genera (*Acostaea* Schltr., *Areldia* Luer, *Cucumeria* Luer, *Empusella* (Luer), *Gerardoa* Luer, *Sarcinula* Luer, *Sylphia* Luer, *Tribulago* Luer, and *Tridelta* Luer). However, the phylogenetic placement of its earliest diverging lineage, *Specklinia* subgenus *Sarcinula*, has very poor support in phylogenies (Karremans et al., 2016) and the author itself later stated that under ‘certain analysis conditions, a closer relationship with *Scaphosepalum* was retrieved’ (Karremans & Vieira‐Uribe, 2020), indicating a tentative inclusion of this group in *Specklinia*. These doubts were later supported by the results of NGS‐based phylogeny of Pleurothallidinae, where *Sarcinula* formed a separate clade from the rest of *Specklinia* (Chumová et al., 2021). However, authors stated that more species should be included in phylogeny prior to the final taxonomic decision.

A specific issue is the classification of a group of four morphologically similar species, showing intermediate characters between *Scaphosepalum* and *Specklinia* (*Sarcinula*): *Specklinia acanthodes*, *S. corniculatum*, *S. medinae*, and *S. rinkei*. These taxa have been assigned to various genera and their close morphological similarity indicating close relationship has been pointed out recently by Karremans and Vieira‐Uribe (2020). In that work, the authors propose the relationship of these taxa with *Sarcinula* clade based on two previous phylogenies. However, in the only available NGS‐based phylogeny of Pleurothallidinae, *S. acanthodes* grouped together with *Scaphosepalum* (Chumová et al., 2021).

The main objective of our study was to look at this clade from multiple methodological perspectives and to determine their relative congruence and potential disagreements in order to gain a comprehensive insight into its evolutionary history. To this end, we conducted a series of whole‐genome, phylogenetic, ecological, and biogeographic analyses. We were able to resolve the phylogeny of this clade at the generic level, identifying its poorly supported parts, including the description of alternative evolutionary scenarios. We have identified a leading role for the Andean region in driving both species diversity and variation in genome‐wide traits.

## RESULTS

### Phylogenetic reconstructions based on the nuclear and plastome DNA

**Figure 1:**
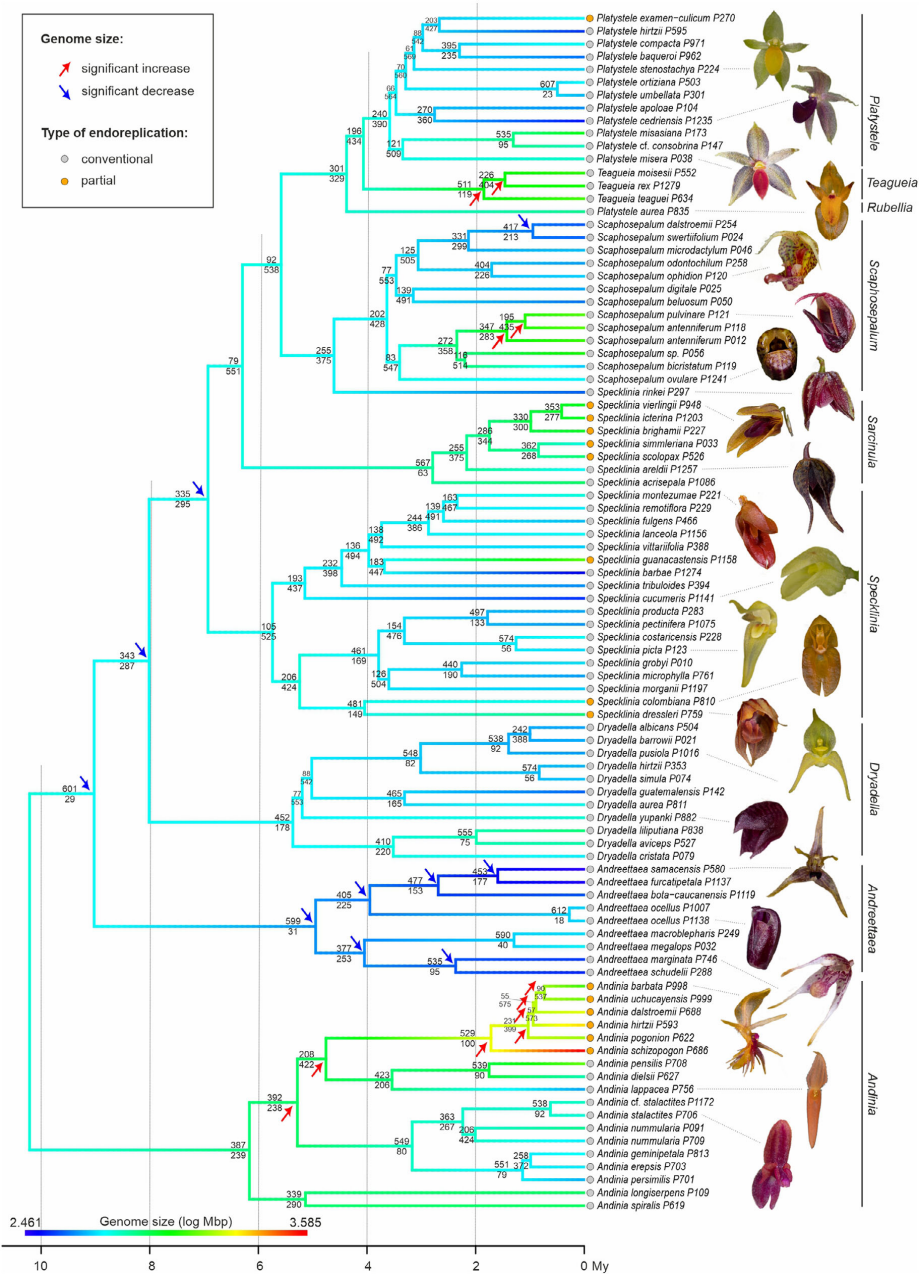
Dated ASTRAL species tree reconstructed from 630 gene trees with visualized evolution of genome size. Lines on the right indicate taxonomical classification at generic level proposed in this paper.

The alignment of the full nDNA dataset is based on 630 loci; the HybSeq statistics (e.g., total number of reads, mapped reads, missing data) are presented in Table S1 for each individual. The ASTRAL phylogeny reflects the division into two main clades—the first clade comprising all members of the genus *Andinia* (labeled as C in Chumová et al., 2021), and the second clade comprising the rest of the genera (labeled as D in Chumová et al., 2021; Figure 1). All other species trees based on various gene selection approaches (see Experimental Procedures section) very well support this division (Figures S1–S4). The PhyParts analysis (Figure S5) provides reasonably good gene tree support for most of the nodes in the ASTRAL species tree (at least 1/3 of concordant gene trees with ASTRAL topology), especially for monophyly of genera. The phylogeny provides very good support for monophyletic genera *Andinia* (387/239 congruent vs. incongruent gene trees), *Andreettaea sensu lato* (599/31) *Dryadella* (452/178), *Sarcinula* (567/63), *Scaphosepalum* (255/375), *Teagueia* (511/119), and *Platystele* (240/390). The only exception seems to be the genus *Specklinia*, with lower support (105 out of 630 gene trees). The cpDNA‐based phylogeny was reconstructed from the alignment of the total length 22 290 bp and the inferred topology is almost identical with the ASTRAL species tree (Figure S6).

### The effect of individual species on tree topology support

Identification of the effect of taxa on topological support was based on removing taxa from all gene trees and recalculating the support of nodes in the ASTRAL species tree after their removal. Key nodes were selected based on lower support and are highlighted in Figure S5 and the results are shown in Table S3. The analysis revealed that removal of *Specklinia rinkei* (= *Scaphosepalum lueri*), a species closely related to the *Scaphosepalum* clade, increased the number of gene trees supporting monophyly of the genus *Scaphosepalum* by 111 (255 with → 366 without). Removal of *Rubellia* (*Platystele*) *aurea* caused a similar effect, increasing support for monophyly of *Teagueia* and *Platystele* by an additional 87 gene trees (301 with → 388 without). This removal also increased *Platystele* monophyly support by 41 gene trees (240 with → 281 without). The relatively low monophyly support of the *Specklinia* clade was only slightly increased by 21 additional gene trees (105 with → 126 without) by removing *Specklinia cucumeris*. Removing additional taxa of the genus *Specklinia* did not further increase the monophyly support of this clade. Although the *Dryadella* clade is well supported at all (452 gene trees show the same topology), there are some discrepancies within it reflected in low support of internal nodes (e.g., N32, Figure S5). Thus, by removing *Dryadella yupanki*, the support of internal node N32 increased slightly by 65 (77 with → 142 without). No other *Dryadella* removal makes such an increase. The synergistic effect of removing all four above‐mentioned taxa affects overall support only marginally, and two nodes in particular (N57 and N64, Figure S5) addressing the relative position of the *Sarcinula*, *Scaphosepalum*, and *Platystele* clades remain with remarkably low support (79/93 and 92/115, respectively; Table S3).

### Variability in species tree reconstruction

In addition to the effects of individual species, we also analyzed species trees based on several approaches to select an appropriate subset of genes for their construction (Figures S1–S4). Our analyses highlighted some specific parts of the phylogeny which are resolved ambiguously.

*Andinia* subgen. *Aenigma* (represented in our phylogeny with *A. barbata*, *A. dalstroemii*, *A. hirtzii*, *A. pogonion*, *A. schizopogon*, *A. uchucayensis*): Trees reconstructed by selection of similar topologies to ASTRAL tree (Figures S1 and S2) indicate existence of two alternative topologies within the genus—first, sister with subgen. *Andinia* (represented here with *A. dielsii*, *A. lappacea*, *A. pensilis*), second, sister with subgen. *Brachycladium* (represented here with *A.* *erepsis*, *A. geminipetala*, *A. nummularia*, *A. persimilis*, *A. stalactites*). However, trees based on ASTRAL monophyly (Figures S3 and S4) naturally indicate sister position to the prevailing signal, that is, to the subgen. *Andinia*. This may indicate hybridogenous origin of the ancestor of this group, which is also reflected in the notable presence of an alternative topology in PieChart analysis (Figure S5, Node N11). Despite the *Andreettaea* clade is very well supported (599 genes with congruent topology; Figure S5), two alternative species trees (Figures S1 and S2) show unambiguous origin of the clade. Some incongruencies with main topologies exist in the genus *Dryadella*. Besides the effect of *D. yupanki*, which is likely to be of a hybridogenous origin (Figures S1, S3, and S4), mutual positions of other internal clades within *Dryadella* are ambiguous. In two trees (Figures S1 and S4), the ancestor of the group of *Dryadella aviceps*, *D. cristata* and *D. liliputiana* has two alternative topologies. In the other species tree (Figure S2), the ancestor of the group of *D. albicans*, *D. barrowii*, *D. hirtzii*, *D. pusiola*, *D. simula* has two alternative topologies.

The *Scaphosepalum* clade is affected by the ambiguous position of *Specklinia rinkei* (see above), but the unclear internal structure of the clade is likely a complex issue. However, the ASTRAL tree reflects the major topology, that is, basal position of *Sp. rinkei*; all alternative species trees show closer affinity of *Sp. rinkei* to the clade containing *Sc. ovulare*, *Sc. bicristatum*, *Sc. pulvinare*, and *Sc. antenniferum* (Figures S1–S4). But in addition to that, there are obviously two alternative times of divergence of this clade from the rest of *Scaphosepalum*. Numerous incongruencies with the main topology given by the ASTRAL tree (Figure S5) exist within the genus *Platystele*; however, these strongly differ between the species trees (Figures S1–S4), which is reflected also in low concordance in topology within the genus (internal nodes of *Platystele* clade, Figure S5). It seems likely that there was a complex ancient hybridization or a rapid radiation that led to incomplete lineage sorting. The topology at the intergeneric level slightly differs across all species trees, namely in the relationship between *Specklinia*, *Sarcinula*, *Scaphosepalum*, and *Platystele* clades, and their ambiguity is reflected by low support of the corresponding nodes in the ASTRAL species tree (Figure S5).

### Testing alternative phylogeny scenarios

As a comparison to the previous approach to analyze alternative topologies in subsets of genes, we also simplified species tree to subsets of taxa representing clades at the level of genera without reduction of number of loci. Based on the ASTRAL species tree reconstruction (Figure S5) we identified monophyletic clades for further testing of their alternative topology, and their list is as follows: *Andinia* (it serves as an outgroup), *Andreettaea s.l*., *Dryadella*, *Specklinia* (subdivided to *Specklinia* 1, *Specklinia* 2, and *Specklinia* 3; see Figure S5), *Sarcinula*, *Scaphosepalum*, *Rubellia*, *Teagueia*, and *Platystele*. Because of the ambiguous topology of two individual taxa (*Specklinia rinkei* [= *Scaphosepalum lueri*] P297 and *Specklinia cucumeris* P1141), we fixed them in all tests for alternative topologies, which resulted in testing trees with 13 tips. Even under this simplification there are (2*k*−3)!!, that is, 3.16 × 10^11^ possible topologies (Rohlf, 1983), if rooted and binary trees with *k* = 13 tips are considered. Further simplification to fully resolved (with 12 nodes) gene trees reduces the number of tested topologies to 184. The top twelve topology scenarios ranked in descending order of support score (average support per node, excluding the root) are shown in Figure S7. The top three topologies differ only in the placement of *Sarcinula*, while the others involve more complex changes. Notable is the occasional placement of *Rubellia* as a sister to *Platystele*, variable placement of *Scaphosepalum* and *Specklinia rinkei* and occasional segregation of *Specklinia* into two distinct clades: (i) *Specklinia* 1 + *Specklinia* 2, and (ii) *Specklinia* 3 + *S. cucumeris*.

### Flow cytometry

**Figure 2:**
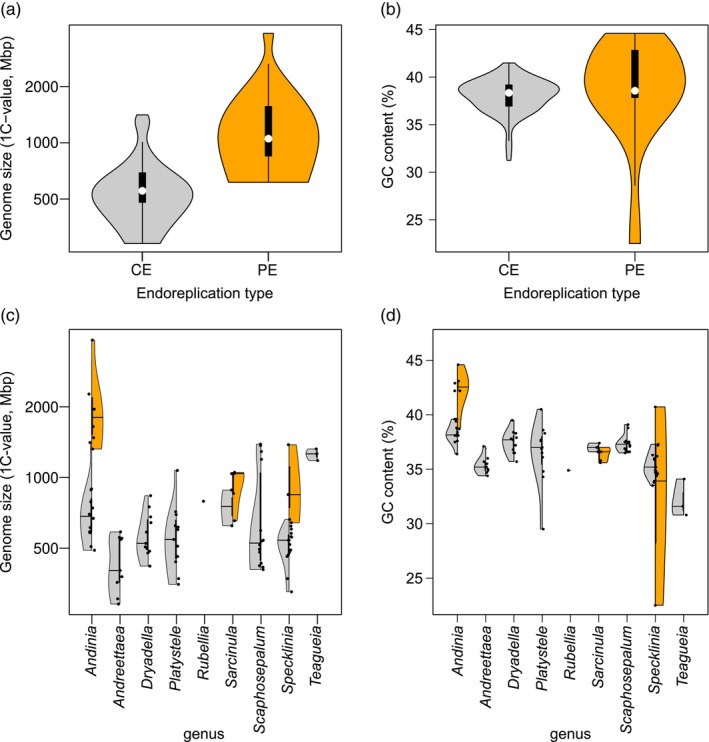
Violin plots of FCM‐derived traits for all species grouped by endoreplication type (a, b) and genus affiliation (c, d). Genome size data are shown in logarithmic scale (a, c) and GC content is expressed as % (b, d). Genus‐level violin plots are shown in two separate halves according to species affiliation to endoreplication type (gray half for conventional and orange half for partial endoreplication).

**Figure 3:**
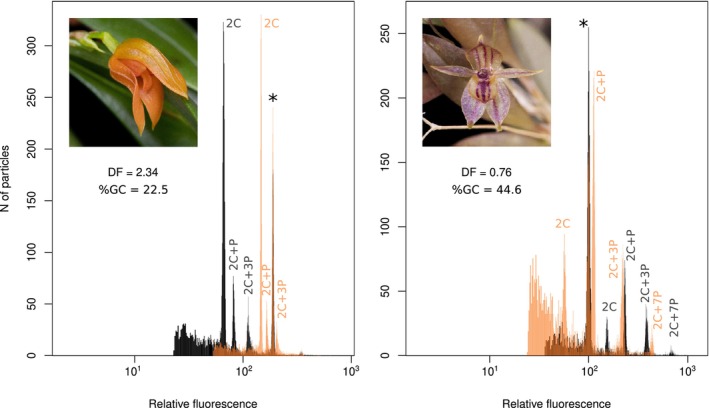
FCM outputs of two species with partial endoreplication (PE) showing two extremes in GC content—*Specklinia guanacastensis* (left pane) and *Andinia dalstroemii* (right pane). Both panels are formed by overlaying log‐scale histograms derived from propidium iodide (PI; black) and DAPI (transparent orange) staining, with the ratio between the relative fluorescence of 2C peaks in the two types of staining being the Dye Factor (DF = DAPI/PI). Individual peaks of both species are labeled according to common practice in PE orchids (e.g., Trávníček et al., 2015), and the standard (*Pisum sativum*) is indicated by asterisks and set to be in superposition for both histograms.

All 93 ingroup individuals and 4 outgroups were subjected to flow cytometry. Genome size varied 13.3‐fold within ingroups, ranging from 289.1 Mbp in *Andreettaea* (*Muscarella*) *samacensis* to 3849.1 Mbp in *Andinia schizopogon* (Table S2). Genome size of nuclei at different stages of endoreplication allowed for the estimation of the type of endoreplication. This analysis showed a prevalence of taxa with conventional endoreplication (CE; 78 individuals) over those with partial endoreplication (PE; 15 individuals). The proportion of the replicated part of the overall genome within PE taxa ranged from 25.5% in *Specklinia guanacastensis* to 85.4% in *Platystele examen‐culicum*. Although the range of genome size variation was highly comparable between CE and PE taxa, both ANOVA and Phylo.ANOVA detected significant differences in average genome size between the two groups (*F*_1,91_ = 48.84, *p*_ANOVA_ <0.001, *p*_PHYLO.ANOVA_ = 0.001; Figure 2a). Such disproportion in genome size is also well documented in genera comprising species with both types of endoreplication (Figure 2c). A significant difference in variation corresponding to the endoreplicated and the non‐endoreplicated part of the genome was found in PE species (3.1‐fold vs. 31.5‐fold variation, respectively). A mutual comparison of the endoreplicated part of genomes in PE and CE taxa (corresponding to the whole genome in the latter) revealed no significant difference (*F*_1,91_ = 1.86, *p*_ANOVA_ = 0.176; *p*_PHYLO.ANOVA_ = 0.470). DNA base composition (GC content) differed by 22.1% between the taxa with the lowest and highest values, *S. guanacastensis* (22.5%; Figure S8) and *Andinia dalstroemii* (44.6%), respectively (Figure 3). Both extremes were estimated in PE species (Figure 2b,d). Summarized data are listed in Table S2. Although the difference in GC content between the CE and PE taxa was significant in the ANOVA (*F*_1,91_ = 4.26, *p*_ANOVA_ = 0.042), phylogenetic correction does not support the difference (*p*_PHYLO.ANOVA_ = 0.255). Highest variation is documented in the genus *Specklinia*, which spans almost both extremes in two species with partial endoreplication—*S. guanacastensis* (22.5%) and *S. dressleri* (43.6%; Figure 2d). Analysis of GC content in nuclei that underwent different numbers of runs of endoreplication (zero, first, and second runs) shows striking shifts in some species with partial endoreplication (Figure S9).

### Genomic traits evolution

Phylogenetic signal of genome size (log transformed 1C‐value in Mbp) was really strong (Pagel's *λ* = 1.0; CI = 0.96–NA) and its projection shows that prevails lower or intermediate GS and almost all clades (at the level of genera) in the phylogeny exhibit a trend of gradual evolution of GS (Figure 1). Significant changes were detected in *Andinia* subgen. *Aenigma* (represented with species *A. schizopogon*, *A. pogonion*, *A. hirtzi*, *A dalstroemii*, *A. uchucayensis*, and *A. barbata*) with a striking increase in GS, associated with the transition to partial endoreplication (Figure 1). At the level of the genus was found significant increase in *Teagueia*. Conversely, a significant decrease in GS was found in the genus *Andreettaea sensu lato* (at least for the ancestral state of the genus and for the 5 nested nodes). Other changes occur on a limited scale and are usually associated with one or a few taxa in the phylogeny and have not been found to be significant (the exception being three individuals of *Scaphosepalum*, Figure 1). Because of the existence of partial endoreplication, we have also analyzed the genome size of the part of the genome which is replicated. This analysis shows very similar results in phylogenetic signal (Pagel's *λ* = 1.0; CI = 0.95–NA) and significant changes in *Andinia*, *Teagueia*, and *Andreettaea* clades (Figure S10). In addition, a significant increase was detected in part of the *Scaphosepalum* clade and a decrease in multiple basal nodes of the *Specklinia* clade (Figure S10). GC content analysis showed a weaker phylogenetic signal (Pagel's *λ* = 0.74; CI = 0.40–0.95) and no apparent trend between and within clades (Figure S11). The exceptions are part of the clades *Andinia* and *Specklinia* and the whole clade *Teagueia*. In the same part of the *Andinia* clade that shows a significant increase in GS, an increase in GC was also found. In addition, an increase was also detected in two other internal nodes in the *Andinia* clade, leading to the detection of a significant increase at the basal node of the whole *Andinia*. Exceptionally low GC content of *S. guanacastensis* caused a significant decrease detected in the appropriate part of *Specklinia* clade (Figure S11).

Analysis of the relationship between GC content and genome size shows only a marginally significant negative correlation (*p*_PGLS_ = 0.058). When the dataset is reduced to orchids with only conventional endoreplication (78 of 93 accessions), the relationship becomes stronger (*p*_PGLS_ = 0.009; Figure S12), whereas the analysis of only orchids with partial endoreplication (15 accessions) lacks any relationship (*p*_PGLS_ = 0.585).

### Spatial analysis of genomic traits

**Figure 4:**
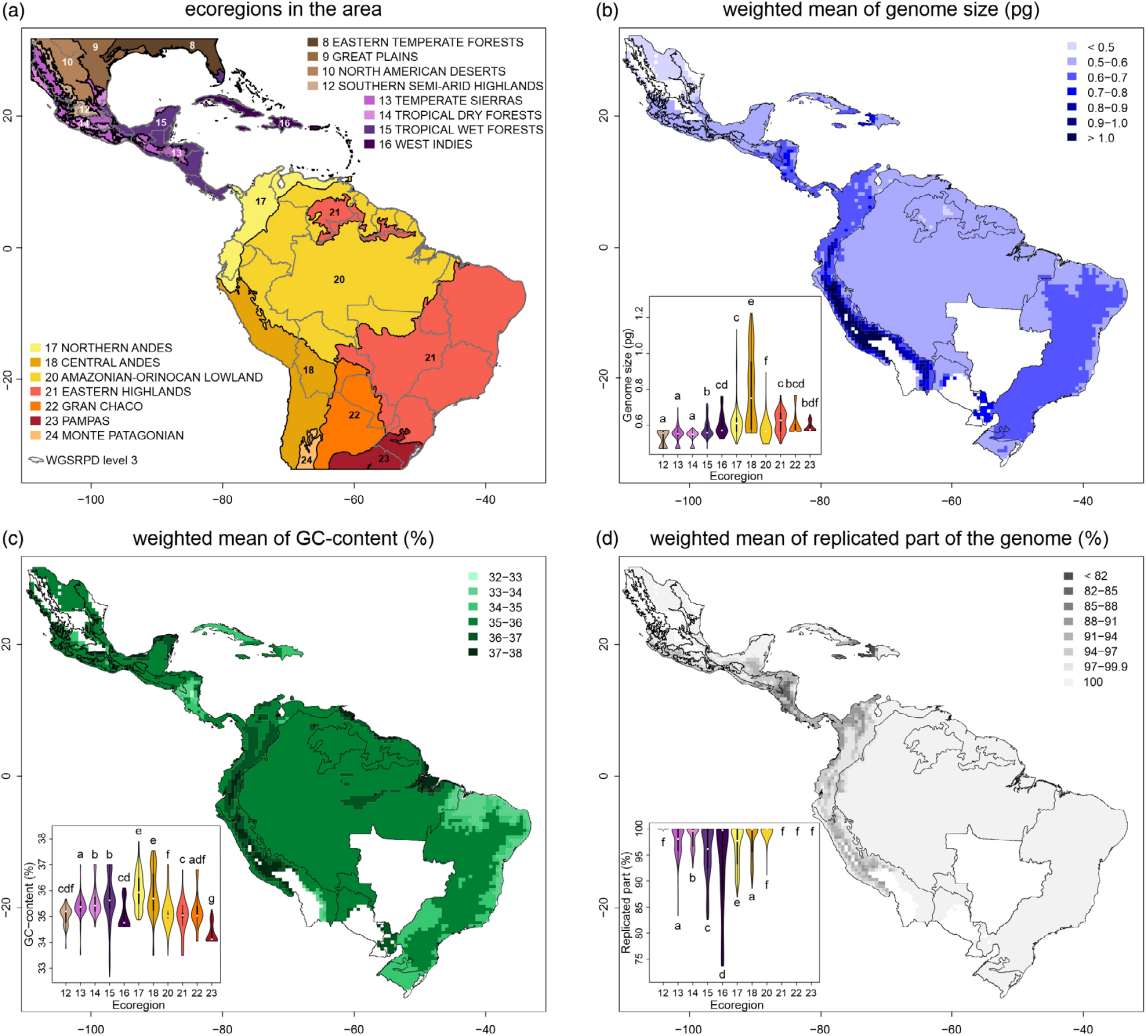
Spatial arrangement of genomic traits inferred from pseudo‐occurrence data aggregated in a 1° arc grid of 85 taxa in the phylogeny with distribution data. Genomic traits for each grid cell correspond to the weighted mean of all taxa co‐occurring in that cell, weighted by their occupied area in the cell. The inset in the graph for each trait represents the differences between ecoregions within the area given by the top left panel. Differences are complemented by clustering based on the TukeyHSD test. Colored areas represent the sum of the 85 taxa ranges included in the spatial analysis.

Spatial analysis of FCM‐related genome traits shows non‐random distribution across the target area (Figure 4). Weighted means of genome traits for artificial grid overlaying the range of all taxa show apparent differences between lowland and mountain areas. For better insight into data structure, grid cells were sorted based on their affiliation to the ecoregions in the area (Figure 4, inlets with violin plots with TukeyHSD comparisons). This analysis points to the fact that genome size is generally higher in mountain regions (ecoregions 17—Northern Andes and 18—Central Andes) and simultaneously shows more variation there. GC content partly follows the same pattern, but the highest variability encounters tropical wet forest (ecoregion 15). The pattern of the proportion of the replicated part of the genome points to the accumulation of species with partial endoreplication in the mountain regions and in regions of Central America and Caribbean islands. The weighted pattern of genomic traits is, of course, dependent on the abundance of species in each cell of the spatial grid, so to reveal the distribution of species in the target area, the species abundance grid was plotted in several ways—total species density, species density for the two endoreplication modes separately, and relative species density as the ratio of PE versus CE species per grid cell (Figure S13).

### Ecological implication for genomic traits

The effect of environmental data (bioclimatic variables) on FCM traits through direct PGLS based on percentile and mean values showed the relationship of genome size with precipitation of the driest month (bio14), seasonality of precipitation (bio15), and precipitation of the driest quarter (bio17, Table 1). All these variables were analyzed in the simple model as separate explanatory variables because more complex models did not show an improvement in explained variability (data not shown). Analysis with allowed variability of the explanatory variables (performed using the sensiPhy approach) showed a significant relationship with bio15 and a marginally insignificant relationship with bio14 when variability was set to the median (i.e., 50% percentile) ± 2.5%. A significant relationship was revealed only with bio15 when variability was increased to ±5% (Table 1; Table S4; Figure S14). In contrast, there was no significant relationship between GC content and any environmental variable (Table 1; Table S4).

**Table 1:** Selected results of PGLS analyses performed on genome size and environmental variables at various levels of statistical aggregation.

Genome size (logGS, Mbp)				
Statistical aggregation	Probability/explained variation	Bioclimatic variables		
		bio14	bio15	bio17
p.10	*p*‐value	0.062	**0.039**	0.114
	expl. var (%)	4.1	**5.0**	3.0
p.25 (Q1)	*p*‐value	**0.024**	**0.014**	**0.046**
	expl. var (%)	**6.0**	**7.1**	**4.7**
p.50 (Q2, median)	*p*‐value	**0.039**	**0.006**	0.054
	expl. var (%)	**5.1**	**8.8**	4.4
p.75 (Q3)	*p*‐value	**0.045**	0.069	**0.043**
	expl. var (%)	**4.8**	3.9	**4.8**
p.90	*p*‐value	**0.044**	0.116	**0.042**
	expl. var (%)	**4.8**	2.9	**4.9**
Mean	*p*‐value	**0.018**	**0.018**	**0.023**
	expl. var (%)	**6.6**	**6.6**	**6.1**
st. dev.	*p*‐value	0.057	0.551	0.063
	expl. var (%)	4.3	0.4	4.1
p.50 ± 2.5%	*p*‐value	0.051 (0.050–0.053)	**0.009** (0.008–0.009)	‐
p.50 ± 5.0%	*p*‐value	0.092 (0.088–0.097)	**0.021** (0.019–0.022)	‐

Bio14, precipitation of the driest month; bio15, precipitation seasonality; bio17, precipitation of the driest quarter. All results are available in Table S4. Bold values indicate *p* ≤ 0.05.

### Biogeography

**Figure 5:**
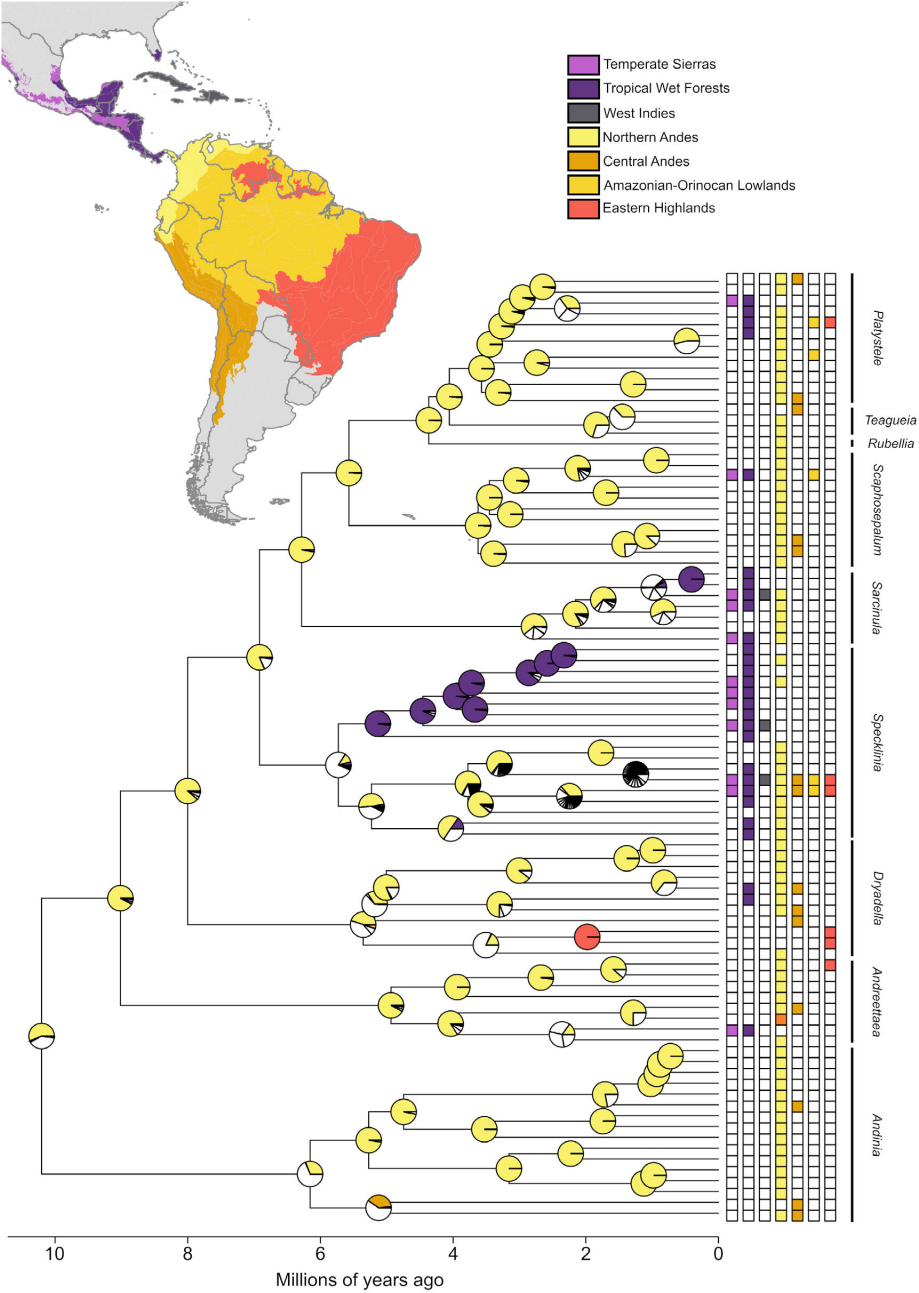
Dated ASTRAL species tree showing the estimated ancestral biogeographic history of the clade D in seven ecoregions throughout Central and South America. Gray areas on the map were not used in the analyses. Pie charts at each node represent ancestral geographic ranges. Colors depicted in the pie charts are those related to the map. White color relates to a combination of two or more regions. Refer to Table S7 for the probability of each area in the main lineages.

Our BioGeoBEARS analyses all inferred the origin of the clade D in the Andes, either the Northern part or a mix of the North and Central parts (Figure 5). We found that the Dispersal–Extinction–Cladogenesis with founder events model was the best‐fitting biogeographical history model (Δ_AICc_ = 405.4, Table S5). The biogeographic events in decreasing order of their percent representation that were observed within the clade D phylogeny were as follows: speciation within‐area (67.6%) followed by range expansions (56.2%) (i.e., range expansion events [54.8%] and founder events [1.38%]) (Tables S6–S10). For dispersal events, the Northern Andes is the highest source of dispersals to other regions (>60%). Usually, the highest receptors of species are the Central Andes and the Tropical Wet Forests in Central America, while the Eastern Highlands and the Amazon‐Orinocan lowlands have the lowest percentages of dispersion into them and reception to them.

## DISCUSSION

Our paper addresses the phylogenetic complexities within the Pleurothallidinae subtribe and highlights significant discordances in gene tree topologies that are likely due to incomplete lineage sorting. Various species tree building methods are used to discern strongly supported clades and identify potential hybrid origins. The evolution of genomic traits, such as genome size and GC content, is examined, along with their correlation with endoreplication types. Spatial trends and biogeography are analyzed to understand the contrasting patterns between Andean areas and other regions. All findings also have important taxonomic implications, supporting the current concept of genera while suggesting adjustments based on phylogenetic and other evidence.

### Phylogenomic perspectives on rapid radiations

The evolutionary history of organisms reveals uneven rates of diversification and speciation over time (e.g., Henao Diaz et al., 2019). Periods of rapid species accumulation have particularly attracted scientific attention for decades (e.g., Sauquet & Magallón, 2018). The primary approach to addressing this phenomenon involves reconstructing phylogeny, followed by macroevolutionary analyses, often complemented by biogeographic studies (in orchids, e.g., Lagou et al., 2024; Pérez‐Escobar et al., 2017, 2024). This approach enables the identification of clades with varying diversification rates in relation to the biogeography of the group under study. Another viable perspective is examining the evolutionary consequences of novel traits, such as changes in diversification tempo associated with trait evolution (in orchids, e.g., Breitkopf et al., 2015; Givnish et al., 2015). Our approach primarily follows the first perspective but expands upon it by incorporating whole‐genome trait analyses, bridging both perspectives. For example, genome size evolution rates have been found to correlate with diversification rates in angiosperms (Puttick et al., 2015) and ferns (Fujiwara et al., 2023). Previous studies on orchids also indicate nonrandom changes in whole‐genome traits along phylogenies (e.g., Leitch et al., 2009; Trávníček et al., 2019), highlighting the utility of integrating such data into complex analyses of relationships among closely related species in species‐rich plant groups (as demonstrated by, e.g., Chumová et al., 2021).

### Phylogeny and conflicting topologies

Our phylogenetic reconstruction using various species tree building method (but mostly relying on ASTRAL‐III approach; Zhang et al., 2018) produces species trees that show strong gene tree support for most nodes, suggesting sister placement of clades C and D and monophyletic origins for most lineages at the genus level. However, some nodes displayed significant discordance in gene tree topologies. Due to the rapid diversification of Pleurothallidinae, many of these phylogenetic incongruities are likely caused by incomplete lineage sorting, a common occurrence in cases of rapid diversification or morphological innovation (Feng et al., 2022; Parins‐Fukuchi et al., 2021). For the purposes of systematic classification, it is essential to discern which clades are strongly supported and which may have a hybrid origin or other sources of evolutionary complexity. To address this, we visualized the discordances and tested our phylogeny for (i) the impact of individual problematic taxa, (ii) the effect of subsetting the ‘best’ gene trees, and (iii) the influence of selecting random subsets of representatives within predefined clades. In addition to using gene tree reduction methods to facilitate computationally intensive coalescent approaches (e.g., gene shopping via the SortaDate approach; Smith et al., 2018), we developed custom scripts to identify prevalent alternative gene‐tree‐based topological scenarios and test their robustness. This combination of methods allowed us to pinpoint lineages with two predominant alternative topologies, suggesting hybrid origins, as was exemplified by the origin of *Andinia* subgen. *Aenigma*, which is likely an ancient hybrid between the ancestors of subgen. *Andinia* and subgen. *Brachycladium*. Further significant discordances between gene trees, possibly indicating hybrid origin, have been found at the intrageneric level within almost all genera, but likely without affecting the topology of the backbone clade(s). This is reflected in relatively low support of main nodes at the level of intrageneric relationships, however, the concordance in backbone topology between different approaches (both in gene tree subsetting and in nuclear‐based versus plastid‐based phylogenies) shows the strong support of the resulting species tree (Figure 1). As we were primarily interested in intergeneric relationships, and species sampling was adjusted to this, we did not identify the sources of phylogenetic conflicts at intrageneric level.

Problematic exceptions are (i) interrelationships between the genera *Sarcinula*, *Scaphosepalum*, and the clade comprising the genera *Platystele*, *Rubellia*, and *Teagueia*, and (ii) interrelationships between genera within the clade comprising *Platystele*, *Rubellia*, and *Teagueia*, which are mostly due to the ambiguous placement of the genus *Rubellia*. It should be noted that analysis based on nuclear ribosomal internal transcribed spacer (ITS) and chloroplast matK sequence data placed *Rubellia* as sister to *Platystele* with low support (Karremans et al., 2016). A considerable amount of topological incongruence is also present in *Specklinia sensu stricto*, which in some trees splits into two main clades, which are located either in the sister position of the genera *Sarcinula and Scaphosepalum* or within the clade comprising genera *Platystele*, *Rubellia*, and *Teagueia*. However, all these alternative topologies were recorded at low frequency in the gene trees, and the major phylogenetic signal that identifies *Specklinia s. s*. as a monophyletic lineage sister to the genera *Sarcinula*, *Scaphosepalum*, *Platystele*, *Rubellia*, and *Teagueia* clearly prevails. On the other hand, the existence of conflicting topologies may explain why an analysis based on ITS and chloroplast matK sequence data placed *Sarcinula* as a sister lineage to *Specklinia s. s*. (Karremans et al., 2016).

Moreover, within the *Specklinia s. s*., three morphologically distinct species exhibit numerous discordances so their position is less supported (*Specklinia colombiana*, *S. cucumeris*, *S. dressleri*). It should be noted that all these three species had been proposed to represent their own genera (*Acostaea*, *Cucumeria*, *Areldia*) within taxa included in our sampling (Luer, 2004). The similar issue applies for the genus *Dryadella*, which is clearly monophyletic, however, there are two species causing internal incongruences (*D. cristata*, *D. yupanki*) and one of these has also been proposed as a representative of its own genus *Incaea* (Luer, 2006). Another separate species is *Specklinia rinkei* (= *Scaphosepalum lueri*) whose assignment to the genus *Scaphosepalum* is clearly the prevailing topology, although the numerous gene trees are contradictory. Likewise, its placement as sister to the rest of the genus shows numerous inconsistencies. Interestingly, all these phylogenetically “problematic” species are morphologically unique and highly divergent from their relatives, which often allows their alternative morphological delimitation as separate genera. The relationship between phylogenetic conflicts and morphological innovations has already been identified at a much deeper phylogenetic level (Parins‐Fukuchi et al., 2021), suggesting that this relationship may be more general at different phylogenetic levels. Somewhat more complex relationships appear to exist within other genera such as *Platystele*, *Teagueia*, and *Scaphosepalum*. However, resolving their internal relationships requires more robust sampling and more detailed research that is beyond the scope of this study.

### Evolution of genomic traits

Flow cytometry provides a unique insight into orchids by unambiguously determining the type of endoreplication, a known unique feature of orchid biology (e.g., Bory et al., 2008; Hřibová et al., 2016; Piet et al., 2022; Trávníček et al., 2019). In addition, it allows us to evaluate the evolution of genome size and GC content, which have been confirmed to be important in many plant groups as well as in orchids (e.g., Chumová et al., 2021; Leitch et al., 2009; Trávníček et al., 2019). In orchids in particular, the interrelationship between endoreplication type and other genomic features is prone to findings that do not occur in other plant groups. For example, exceptional variability in genomic GC content is tightly associated with partial endoreplication, as was corroborated in this study (Figure S11) as well as in previous studies (Chumová et al., 2021; Trávníček et al., 2019). Interestingly, the minimum GC content found here in *S. guanacastensis* (22.5%, Figure 2) represents a newly established lower limit for plants and is really close to known minimum for any eukaryotic organisms (*Plasmodium falciparum*, GC = 19%; Gardner et al., 2002). In addition, the variability in GC content in the group of orchids studied is negatively correlated with genome size when only plants with conventional endoreplication are considered (Figure S11). This finding contrasts with several studies that found a positive correlation with monoploid genome size (Bureš et al., 2024; Lipnerová et al., 2013; Šmarda et al., 2019; Veleba et al., 2014, 2017). A possible explanation for such a pattern is the role of GC‐rich repetitive DNA, which serves as a driving force in genome enlargement or shrinkage (e.g., Stritt et al., 2020). In orchids, however, it acts in a different direction than is usually observed in grasses (Estep et al., 2013; SanMiguel & Vitte, 2009) or Asteraceae (Olanj et al., 2015). An indirect view of the proportion of GC content in repetitive sequences is provided by analysis of GC content in all subsequent peaks in orchids with partial endoreplication (Figure S9). With respect to the partial or complete elimination of repetitive DNA during partial endoreplication (Chumová et al., 2021; Piet et al., 2022), a comparison of the relative increase or decrease in GC content between fractions of nuclei undergoing different numbers of cycles of endoreplication provides a powerful tool to reveal the theoretical base composition of repetitive DNA. Clearly, if there is a shift away from a balanced AT/GC content ratio in the orchids examined, it is toward a higher proportion of GC content in repetitive DNA (Figure S9). The one and only notable exception is the apparently GC‐poor repetitive DNA in *S. guanacastensis*, where there is a striking increase in the proportion of GC in nuclei with reduced influence of repetitive elements, that is, from differentiated cells (Figure S9). Thus, species with PE generally exhibit extreme values in GC content likely due to accumulation of repetitive DNA with unbalanced GC proportion (Chumová et al., 2021). Surprisingly, the genus *Sarcinula* is an exception from this relationship because *Sarcinula* species with PE are conserved in GC content. This genus seems to behave somewhat differently in evolution as indicated by the highly variable size of the endoreplicated part of the genome exhibiting 47% difference within PE species of the genus. In other orchid PE species, the size of endoreplicated part of the genome is conserved in genera or at least in evolutionary lineages of closely related species (Chumová et al., 2021; Trávníček et al., 2021). Thus, it can be inferred that genome evolution may differ between lineages, and upcoming research is likely to reveal different mechanisms by which genomes with PE may be maintained.

It is also worth noting that observed rates of species differentiation remain relatively stable when comparing PE versus CE lineages (see Supporting Information). The persistence of consistent speciation rates suggests that factors beyond endoreplication may play pivotal roles in shaping the evolutionary trajectories of these species (Šmarda et al., 2014), warranting further investigation into the intricate interplay between genomic dynamics, ecological factors, and evolutionary forces.

If we compare our data with available chromosome counts (Chumová et al., 2021; de Oliveira et al., 2015), there is generally no relationship between genome size and number of chromosomes. The chromosome numbers seem to be more conserved at the level of genera. For example, one lineage within *Scaphosepalum* increased GS significantly (Figure S9), but not the chromosome number which is stable in the whole genus (2*n* = 28–32; Chumová et al., 2021).

### Spatial trends and biogeography

The spatial analysis of the data revealed distinct patterns between the Andean regions and other areas inhabited by the study species, with remarkable differences observed in the distribution of changes among all tested genome‐wide traits (Figure 4). Notably, the changes in genome size closely mirrored the distribution of the replicated portion of the genome, highlighting a strong relationship between these two traits. Accordingly, the largest genome sizes and the smallest fraction of replicated genomes accumulate in the Central Andes and other regions associated with the Andean uplift. Patterns of variability in GC content are more patchy, with the greatest contrast between Andean areas, along with small ranges along the northern coast and the westernmost parts of South America, along with some parts of Central America and the Caribbean (Figure 4). It is likely that some of the observed patterns are related to the species richness associated with the Andean uplift (Lagomarsino et al., 2016; Pérez‐Escobar et al., 2017), that is, higher spatial species richness tends to reflect higher variability in genomic traits, although the genome size *per se* is not related to the increasing rate of speciation in the clade D (Supplementary Material). Moreover, the high contribution of lineages derived from the Andes to Central America and the Caribbean, as predicted by our stochastic mapping analysis, is possibly a strong predictor of this extant pattern (Figure 5; Table S5).

On the other hand, some trends may be attributed to differences in species distribution patterns and intrinsic characteristics—for example, the higher genome size associated with the Central Andes is related to the distribution pattern of *Andinia s. l*., the genus carrying the highest genome size among the plants studied here. Surprisingly, species of a clade D with PE are absent in the east of the Andes (Figure 4d; Figure S13). This could be more general in Pleurothallidinae because the majority of genera where PE has been recorded (e.g., *Brachionidium*, *Dracula*, *Masdevallia*, *Porroglossum*, *Zootrophion*; Chumová et al., 2021) are generally absent or poorly represented in the east of the Andes, with the exception of the genus *Anathallis*, which is widely distributed in Eastern Brazil. This disjunct biogeographic pattern observed between the Andes and the mountains in eastern South America has been previously acknowledged in various studies (e.g., Bochorny et al., 2022; Chaves et al., 2015; Dantas‐Queiroz et al., 2023; DeForest Safford, 2007). These studies explore the evolutionary history of these mountain ranges in connection to dispersal events through ancestral corridors, such as the Patagonian‐Chacoan region, potentially linking the two extremes of the South American continent (Chaves et al., 2015; Fiaschi & Pirani, 2009). Nonetheless, the biogeographic history of the clade shows us that few founder events outgoing from the Andes (the putative center of origin and diversification of this clade—Figure 5; Tables S5 and S10) explain why there are so few species with PE in Extra‐Andine areas. Many genera with PE are predominantly distributed at higher altitudes, so it could be hypothesized that their preference for mountainous environments could be responsible for their underrepresentation east of the Andes, although some lineages or species with PE occur in lowlands west of the Andes, for example *Sarcinula* and some species of *Specklinia*. This spatial structure is supported by correlations between some bioclimatic variables and observed genome‐wide traits. Specifically, genome size was found to be positively correlated with seasonality of precipitation (bio15) and negatively correlated with precipitation of the driest month (bio14; Figure S14). Both variables indicate that species with larger genome sizes are able to inhabit sites with greater variation in precipitation throughout the year. A similar trend, but on a larger and coarser scale, has been found for whole orchids, where tropical representatives (species likely prone to grow in precipitation‐stable conditions) tend to have smaller genomes than others (Trávníček et al., 2019). In general, the response of plant genome size to the precipitation gradient varied significantly among the groups studied—for example, Patagonian representatives of the genus *Berberis* showed a positive correlation between genome size and annual precipitation (Bottini et al., 2000), whereas a negative correlation was found in the genus *Pinus* (Grotkopp et al., 2004).

### Taxonomic implications

Our results mostly support the current concept of genera as monophyletic lineages. A monospecific genus *Rubellia*, which is sometimes included in *Plastystele*, is well separated from the genus *Teagueia*, corroborating our previous results (Chumová et al., 2021). All three mentioned genera form a relatively young monophyletic group that is highly variable in genome size, but share chromosome number 2*n* = 26 (Chumová et al., 2021), so they could be alternatively merged into a single genus *Platystele*. However, such a taxonomic change is not necessary; all three genera are morphologically easily distinguishable, and *Teagueia* has generally larger genomes than *Platystele* and *Rubellia*, so we are keeping these genera separate. The morphologically unique species *D. yupanki*, sometimes separated as a genus *Incaea*, was found to be nested in *Dryadella*, corroborating previous results of Karremans et al. (2016). Our results also unequivocally support the broad concept of the genus *Andreettaea*, which was proposed relatively recently (Doucette, 2022; Doucette et al., 2022). Sequencing of nuclear ribosomal internal transcribed spacer and chloroplast matK placed *A. ocellus* within *Muscarella* (Doucette et al., 2022), so both genera were taxonomically merged under *Andreettaea*, which has a priority (Doucette, 2022). Our results also place *Andreettaea* within the lineage of *Muscarella*, so we can confirm this taxonomic hypothesis with more robust data. The genus *Andreettaea* in its broad sense seems to be characterized by generally smaller genomes. The unique flower morphology of *A. ocellus* is likely an adaptation to a specific, yet unknown, pollination mechanism distinct from that of other species formerly classified as *Muscarella*.

However, we found some interesting discrepancies with the former generic concept of the genus *Specklinia*. This genus is polyphyletic, separated into three main lineages. One lineage corresponds to the former *Specklinia* subgenus *Sarcinula* (Karremans et al., 2016) except of the species with non‐resupinate flowers which form another lineage (represented by *Sp. rinkei* in our analyses; the same sample misidentified as *Scaphosepalum medinae* in Chumová et al., 2021). All the rest of the species of *Specklinia* is grouped in the third, largest and heterogeneous lineage. Phylogeny of *Specklinia* has been thoroughly assessed by Karremans et al. (2016) based on nuclear ribosomal internal transcribed spacer and chloroplast matK sequence data. They nicely delimitated the *Specklinia* subgenus *Sarcinula* lineage, however its placement as the earliest diverging lineage of *Specklinia* was poorly supported and the author itself later stated that under ‘certain analysis conditions, a closer relationship with *Scaphosepalum* was retrieved’ (Karremans & Vieira‐Uribe, 2020). Our first phylogenetic reconstruction of Pleurothallidinae based on multiple low‐copy nuclear gene sequencing approach (Chumová et al., 2021) clearly separated *Sarcinula* from *Specklinia*, but the number of sampled taxa was low. Here we supported the separation of *Sarcinula* from *Specklinia* on a significantly larger number of taxa. It should also be noted that *Sarcinula* has been firstly proposed at a generic level by Luer (2006). Nevertheless, his treatment was much broader than that of Karremans et al. (2016) and that of this study, and it also included species, which in fact belong to the core *Specklinia*. Although the independent position of *Sarcinula* is clear, its placement in the topology is somewhat tricky, as it is shown by alternative topology scenarios (Figure S7). The majority signal placed *Sarcinula* as a sister group to a clade comprising *Scaphosepalum*, *Rubellia*, *Platystele*, and *Teagueia*; the second most likely scenario placed *Sarcinula* with *Scaphosepalum* as a sister clade to a common clade comprising *Rubellia*, *Platystele*, and *Teagueia*; and the third most likely scenario creates a topology of descendants beginning with *Scaphosepalum*, *Sarcinula*, *Rubellia*, and *Platystele* with *Teagueia* (Figure S7). In all of these scenarios, the rest of the genus *Specklinia* forms another sister clade that does not interfere with the placement of the *Sarcinula* lineage.

The second lineage, represented in our sampling by *Sp. rinkei*, consists of four morphologically similar species (*Specklinia acanthodes*, *Sc. corniculatum*, *Sp. medinae*, and *Sp. rinkei*). They were assigned to different genera, mainly *Scaphosepalum* and *Specklinia*. Karremans and Vieira‐Uribe (2020) proposed the relationship of these taxa to the clade *Sarcinula* based on two previous phylogenies. Although the first work of Endara (2013) used several loci (ITS, matK, trnL, Xdh, and ycf1), his individual trees generally have low support, and three species of this lineage were placed at different positions within the genera *Scaphosepalum* or *Specklinia*. The second work of Karremans et al. (2016) reportedly used *Sp. acanthodes* (as listed in the accessions analyzed), but it is not present in any trees. Nevertheless, its taxonomic position was determined to be *Specklinia* subgenus *Sarcinula*, although no data pointed to this outcome. The first sequence data indicating the grouping of this lineage with *Scaphosepalum* was obtained in our previous work (Chumová et al., 2021), but we did not address this issue in detail. Therefore, the phylogenetic placement of this lineage has been highly speculative until now, and our results provide the first evidence‐based proposal for its placement as sister to the genus *Scaphosepalum*. As already mentioned, all four members of this lineage are morphologically very close to each other (Karremans & Vieira‐Uribe, 2020) and they likely represent a single evolutionary lineage. They can be easily differentiated from *Specklinia* and *Sarcinula* by the non‐resupinated flowers which bear the lip in the upper position and the presence of at least small cushions on lateral sepals. These features are shared with the rest of *Scaphosepalum*. There is also one species morphologically intermediate between these taxa and *Scaphosepalum s. s*., *Scaphosepalum pleurothallodes*. We were unable to analyze this species, but it shares more characters with the above‐mentioned four taxa than with the rest of *Scaphosepalum*, so it would likely also be a member of this group. If so, this entire group can be easily differentiated from other *Scaphosepalum* species by the lip without lamellae and sepals not distinctly elongated into tails. Taking into account the phylogenetic placement of *S. rinkei* in our phylogenies, we propose to classify all these species as a separate subgenus in *Scaphosepalum*.

The third lineage of *Specklinia* represents the rest of the genus, which is morphologically variable. It is likely monophyletic but it splits into two main lineages whose monophyly is much better supported than the monophyly of the whole genus. Divergence of both lineages is relatively old and one of them likely evolved in different ecoregion, indicating ancient dispersal of one ancestor to Central America. Considering also morphological differences between both lineages, it could be proposed to separate them at generic level. However, such a taxonomic change is not necessary and keeping both lineages in a single genus seems more practical.

Our study did not focus on a clade C comprising the genus *Andinia sensu lato*. However, our results bring some interesting outcomes with taxonomic implications, especially in relation to the recent article of Szlachetko et al. (2022) who proposed splitting this genus into nine small genera. We did not sample representatives of all these small genera, so the genera *Minuscula*, *Lueranthos*, and *Xenosia* are missing from the results. The rest of the proposed genera are supported as monophyletic in our study, and because the whole group is relatively old, some of them can be alternatively treated as separate genera. The salient example is *Aenigma* (*Andinia* subgen. *Aenigma*), comprising the crown clade of *Andinia s. l*. with really high support (529 out of 630 gene trees) and all members of *Andinia s. l*. with partial endoreplication, increased genome size (Figure 1) and GC content (Figure S10). This lineage may be of hybridogenous origin (between the ancestors of two lineages, one containing *Neooreophilus* and second *Chicalia* + *Andinia s. s*.) as indicated by alternative topologies (Figures S1 and S2). Nevertheless, it is likely not a result of allotetraploidization, as indicated by the available chromosome counts (Chumová et al., 2021) and the size of the evolutionary conserved endoreplicated part of the genome, which is significantly smaller than that of species from sister lineages. However, separation of some other genera is questionable due to their morphological similarity and relatively young evolutionary origin. Separation of the genus *Chicalia* (represented here by *A. lappacea*) from the genus *Andinia s. s*. (represented by *A. dielsii*, *A. pensilis*) makes little sense, as they form a monophyletic lineage, their divergence is relatively young, and they share the morphological features that easily separate them from the rest of *Andinia* (Karremans & Vieira‐Uribe, 2020). Thus, we propose following the classification of Wilson et al. (2017) who placed *A. lappacea* (the single representative of *Chicalia*) in the same taxonomic group with *Andinia s. stricto*, the subgen. *Andinia*. Similarly, separation of *Masdevalliantha* (represented here by *A. longiserpens*) from *Xenosiella* (represented by *A. spiralis*) at the generic level can be considered taxonomically unsupported because both groups form a monophyletic lineage and share numerous morphological characters that can easily distinguish them from the rest of the genus (Karremans & Vieira‐Uribe, 2020). We did not sample *Xenosia*, but it is morphologically practically indistinguishable from *Masdevalliantha*, so both should be very likely treated in the single taxonomic group. Thus, we propose following the classification of Wilson et al. (2017) who placed representatives of all these groups in the same taxonomic group subgen. *Masdevalliantha*. Nevertheless, we do not propose corresponding taxonomic changes at the generic level here, because before testing the phylogenetic placement of *Minuscula* and *Lueranthos* with robust sequence data, this would likely be premature.

#### Taxonomy

***Sarcinula*** Luer, Monogr. Syst. Bot. Missouri Bot. Gard. 105: 201 (2006).

TYPE: *Pleurothallis acicularis* Ames & C. Schweinf., Schedul. Orchid. 10: 21 (1930).

Recent circumscription of this genus includes 17 species that are all listed below:

*Sarcinula acicularis* (Ames & C. Schweinf.) Luer, Monogr. Syst. Bot. Missouri Bot. Gard. 105: 202 (2006).

Bas. *Pleurothallis acicularis* Ames & C. Schweinf., Schedul. Orchid. 10: 21 (1930).

Syn. *Specklinia acicularis* (Ames & C. Schweinf.) Pridgeon & M.W. Chase, Lindleyana 16: 256 (2001).

Syn. *Pleurothallis dixiorum* Luer & Béhar, Lindleyana 6: 97 (1991).

*Sarcinula acoana* (Bogarín) J. Ponert, *comb. nov*.

Bas. *Specklinia acoana* Bogarín, Lankesteriana 13: 188 (2014).

*Sarcinula acrisepala* (Ames & C. Schweinf.) Luer, Monogr. Syst. Bot. Missouri Bot. Gard. 105: 203 (2006).

Bas. *Pleurothallis acrisepala* Ames & C. Schweinf., Schedul. Orchid. 8: 22 (1925).

Syn. *Specklinia acrisepala* (Ames & C. Schweinf.) Pridgeon & M.W. Chase, Lindleyana 16: 256 (2001).

*Sarcinula alexii* (A.H. Heller) Luer, Monogr. Syst. Bot. Missouri Bot. Gard. 105: 204 (2006).

Bas. *Pleurothallis alexii* A.H. Heller, Phytologia 14: 8 (1966).

Syn. *Specklinia alexii* (A.H. Heller) Pridgeon & M.W. Chase, Lindleyana 16: 256 (2001).

*Sarcinula areldii* (Luer) Luer, Monogr. Syst. Bot. Missouri Bot. Gard. 105: 204 (2006).

Bas. *Pleurothallis areldii* Luer, Selbyana 2: 383 (1978).

Syn. *Specklinia areldii* (Luer) Pridgeon & M.W. Chase, Lindleyana 16: 256 (2001).

*Sarcinula barbelifera* (Karremans, Salguero & Bogarín) J. Ponert, *comb. nov*.

Bas. *Specklinia barbelifera* Karremans, Salguero & Bogarín, Phytotaxa 447: 20 (2020).

*Sarcinula berolinensis* (Bogarín) J. Ponert, *comb. nov*.

Bas. *Specklinia berolinensis* Bogarín, Lankesteriana 13: 191 (2014).

*Sarcinula brighamella* (Luer) Luer, Monogr. Syst. Bot. Missouri Bot. Gard. 105: 206 (2006).

Bas. *Pleurothallis brighamella* Luer, Monogr. Syst. Bot. Missouri Bot. Gard. 76: 171 (1999).

Syn. *Specklinia brighamella* (Luer) Pridgeon & M.W. Chase, Lindleyana 16: 256 (2001).

*Sarcinula brighamii* (S. Watson) Luer, Monogr. Syst. Bot. Missouri Bot. Gard. 105: 206 (2006).

Bas. *Pleurothallis brighamii* S. Watson, Proc. Amer. Acad. Arts 23: 285 (1888).

Syn. *Specklinia brighamii* (S. Watson) Pridgeon & M.W. Chase, Lindleyana 16: 256 (2001).

*Sarcinula calderae* (Luer) Luer, Monogr. Syst. Bot. Missouri Bot. Gard. 105: 207 (2006).

Bas. *Pleurothallis calderae* Luer, Orquideologia 22: 53 (2001).

Syn. *Specklinia calderae* (Luer) Luer, Monogr. Syst. Bot. Missouri Bot. Gard. 95: 259 (2004).

*Sarcinula condylata* (Luer) Luer, Monogr. Syst. Bot. Missouri Bot. Gard. 105: 208 (2006).

Bas. *Pleurothallis condylata* Luer, Selbyana 3: 80 (1976).

Syn. *Specklinia condylata* (Luer) Pridgeon & M.W. Chase, Lindleyana 16: 257 (2001).

*Sarcinula icterina* (Bogarín) J. Ponert, *comb. nov*.

Bas. *Specklinia icterina* Bogarín, Lankesteriana 13: 201 (2014).

*Sarcinula purpurella* (Luer) Luer, Monogr. Syst. Bot. Missouri Bot. Gard. 105: 217 (2006).

Bas. *Pleurothallis purpurella* Luer, Monogr. Syst. Bot. Missouri Bot. Gard. 76: 176 (1999).

Syn. *Specklinia purpurella* (Luer) Pridgeon & M.W. Chase, Lindleyana 16: 259 (2001).

*Sarcinula scolopax* (Luer & R. Escobar) Luer, Monogr. Syst. Bot. Missouri Bot. Gard. 105: 217 (2006).

Bas. *Pleurothallis scolopax* Luer & R. Escobar, Orquideologia 14: 172 (1981).

Syn. *Specklinia scolopax* (Luer & R. Escobar) Pridgeon & M.W.Chase, Lindleyana 16: 259 (2001).

*Sarcinula simmleriana* (Rendle) Luer, Monogr. Syst. Bot. Missouri Bot. Gard. 105: 218 (2006).

Bas. *Pleurothallis simmleriana* Rendle, J. Bot. 38: 274 (1900).

Syn. *Specklinia simmleriana* (Rendle) Luer, Monogr. Syst. Bot. Missouri Bot. Gard. 95: 263 (2004).

Syn. *Pleurothallis periodica* Ames, Schedul. Orchid. 7: 21 (1924).

Syn. *Specklinia periodica* (Ames) Pridgeon & M.W. Chase, Lindleyana 16: 258 (2001).

*Sarcinula striata* (H. Focke) Luer, Monogr. Syst. Bot. Missouri Bot. Gard. 105: 219 (2006).

Bas. *Pleurothallis striata* H. Focke, Tijdschr. Natuurk. Wetensch. Kunsten 4: 63 (1851).

Syn. *Humboltia striata* (H. Focke) Kuntze, Revis. Gen. Pl. 2: 668 (1891).

Syn. *Specklinia striata* (H. Focke) Luer, Monogr. Syst. Bot. Missouri Bot. Gard. 95: 264 (2004).

*Sarcinula tirimbina* (Karremans, Salguero & M. Cedeno) J. Ponert, *comb. nov*.

Bas. *Specklinia tirimbina* Karremans, Salguero & M. Cedeno, Phytotaxa 447: 27 (2020).

*Sarcinula vierlingii* (Baumbach) J. Ponert, *comb. nov*.

Bas. *Specklinia vierlingii* Baumbach, Orchidee (Hamburg) 63: 405 (2012).

***Scaphosepalum***

*Scaphosepalum* subgen. *Acanthodes* J. Ponert, *subgen. nov*.

Type: *Pleurothallis acanthodes* Luer, Selbyana 1: 222 (1975).

This subgenus includes five species listed below:

*Scaphosepalum acanthodes* (Luer) J. Ponert, *comb. nov*.

Bas. *Pleurothallis acanthodes* Luer, Selbyana 1: 222 (1975).

Syn. *Sarcinula acanthodes* (Luer) Luer, Monogr. Syst. Bot. Missouri Bot. Gard. 105: 202 (2006).

Syn. *Specklinia acanthodes* (Luer) Pridgeon & M.W. Chase, Lindleyana 16: 256 (2001).

*Scaphosepalum corniculatum* Vierling, Orchidee, Taxon. Mitt. 4(14): 98 (2018).

*Scaphosepalum corniculatum* was synonymized with *S. acanthodes* by Karremans and Vieira‐Uribe (2020), however, both taxa differ with numerous characters and some of these differences are significantly greater than the differences between other species in this group. *Scaphosepalum corniculatum* has for example much larger leaves (24–40 mm long vs. 6–13 mm long, including petiole), longer penducle (26–40 mm vs. 20–35 mm), larger flowers (dorsal sepal 6.8 mm long versus 4 mm, petals 2.6 mm long vs. 1.7–2 mm, lip 3 mm long vs. 2 mm). Thus, we recognize both as separate taxa.

*Scaphosepalum medinae* Luer & J. Portilla, Monogr. Syst. Bot. Missouri Bot. Gard. 79: 131 (2000).

Syn. *Specklinia medinae* (Luer & J. Portilla) Karremans, *nom. inval*.

The name *Specklinia medinae* (Luer & J. Portilla) Luer, Monogr. Syst. Bot. Missouri Bot. Gard. 95: 262 (2004) has already been used for another taxon, *Pleurothallis medinae* Luer & J.Portilla, Monogr. Syst. Bot. Missouri Bot. Gard. 79: 110 (2000), which makes the later combination used for this taxon an illegitimate synonym.

*Scaphosepalum pleurothallodes* Luer & Hirtz, Monogr. Syst. Bot. Missouri Bot. Gard. 44: 126 (1992).

*Scaphosepalum lueri* J. Ponert, *nom. nov*.

Replaced synonym: *Sarcinula rinkei* Luer, Selbyana 30: 18 (2009).

Syn. *Specklinia rinkei* (Luer) J.M.H.Shaw, Orchid Rev. 122: 77 (2014).

The name *Scaphosepalum rinkei* Luer & Endara Harvard Pap. Bot. 16: 348 (2011) has been already used for another taxon, so it cannot be used for this species. Thus, we propose to name this species in honor of Dr. Luer, who originally described it.

## EXPERIMENTAL PROCEDURES

### Taxon sampling

Although our study focuses primarily on clade D according to Chumová et al. (2021), the close proximity of clade C (comprising the single genus *Andinia*; Chumová et al., 2021; Pérez‐Escobar et al., 2017) led us to include both clades and to address them simultaneously. However, the position of the two clades, which always remain sister clades within the rest of the subtribe Pleurothallidinae, may differ when nuclear or chloroplast data are used (e.g., Chumová et al., 2021). We used the corresponding dataset from our previous paper (Chumová et al., 2021; where clade D comprises 48 and clade C 10 specimens), and we added 35 new specimens (of which 27 within cladeD and 8 within clade C) to better cover the diversity of clade D with main focus on the genus *Specklinia sensu lato* (Table S1). We also aimed to sample *Andreettaea*, a genus that was monospecific without sequence data until the recent publication of Doucette et al. (2022). However, the availability of plant material was limited due to the need for live material to measure genomic traits, so the range of samples collected was limited to those growing in European botanical gardens or private collections. It could be argued that our sample is rather low; however, we aimed to cover the geographical ranges of all genera equally, and we tested if our sample is equivalent with the overall distribution of species of target genera. The chi‐square test has shown proportional distribution of our sample against the overall distribution (Table S11).

### DNA extraction

Total genomic DNA was extracted from 0.5 g of silica‐dried leaf material by the sorbitol method (Štorchová et al., 2000) with two modifications: 1600 μL of extraction buffer was used per sample (instead of 1300 μL) and 6 μL of RNase was used instead of 4 μL in the first step. The quality of the DNA was checked on 1% agarose gels and using a Qubit 2.0 fluorometer (Invitrogen, Carlsbad, CA, USA).

### HybSeq library preparation

For each sample, genomic DNA was fragmented using an M220 Focused‐ultrasonicator (Covaris, Woburn, MA, USA; settings 52 sec, 6°C, 200 cycles), and ~800 bp fragment length was verified using agarose electrophoresis. Library preparation followed the NEBNext Ultra DNA Library Prep Kit for the Illumina protocol (New England Biolabs, Ipswich, MA, USA) with the following modifications. Half volumes of samples and NEBNext chemicals were used, an additional cleanup step after the adapter ligation was done using a QIAquick Purification Kit (QIAGEN, Venlo, Netherlands), and size selection to approximately 400–600 bp was performed using Agencourt AMPure XP beads (Beckman Coulter, Danvers, Massachusetts, USA). Amplification of the ligated, size‐selected fragments was done using NEBNext Multiplex Oligos for Illumina Dual Index Primers Set 1 (New England Biolabs, Ipswich, MA, USA) and KAPA HiFi HotStart ReadyMix PCR Kit (Kappa Bioscience, Oslo, Norway). Purification of enriched PCR products was done twice using Agencourt AMPure XP beads at a 0.75 volume ratio.

In‐solution sequence capture of 4956 exons from 1200 nuclear low‐copy genes (Chumová et al., 2021) was done using MYbaits custom probes (MYcroarray, Ann Arbor, MI, USA) following the manufacturer's protocol with a hybridization time of 26 h, 12 cycles of PCR enrichment using KAPA HiFi HotStart DNA Polymerase (ThermoFisher Scientific, Wilmington, DE, USA), purification with the QIAquick PCR Purification kit (Qiagen, Venlo, Netherlands) and quantification using Qubit 2.0 (Invitrogen, Carlsbad, CA, USA).

The samples were sequenced on an Illumina (San Diego, California, USA) NovaSeq 6000 at IAB (Olomouc, Czech Republic) or in Macrogen, Inc. to obtain 150 bp paired‐end reads.

### Processing of raw reads and species tree reconstruction

We used the HybPhyloMaker v.1.6.4 pipeline (Fér & Schmickl, 2018) for raw read filtering, mapping of the filtered reads to a pseudoreference (exon sequences used for probe design, separated by strings of 400Ns) and construction of gene alignments. PhiX reads were removed with Bowtie 2 (Langmead & Salzberg, 2012), SAMtools (Li et al., 2009) and bam2fastq (https://gsl.hudsonalpha.org/information/ software/bam2fastq). Adapter trimming and quality filtering was done using Trimmomatic (Bolger et al., 2014), and FastUniq (Xu et al., 2012) was used for duplicate read removal. Mapping of filtered reads to a pseudoreference was performed using BWA (Li & Durbin, 2009). A consensus sequence by majority rule consensus (majority threshold of 0.6) was generated with Kindel (https://github.com/bede/kindel) and was then compared to the original exon sequences for each sample using BLAT (Kent, 2002) with a minimum sequence identity of 85. Consensus sequences were aligned using MAFFT 7 (Katoh & Standley, 2013) with default settings and sequences from nuclear exons of the same gene assembly were concatenated into the particular genes using AMAS (Borowiec, 2016). Also, gene trees and the species tree were reconstructed according to the HybPhyloMaker pipeline. Gene alignments with more than 30% of missing data and less than 100% species presence were excluded from further analyses. Alignment characteristics were calculated using AMAS (Borowiec, 2016), trimAl 1.4 (Capella‐Gutiérrez et al., 2009), and MstatX (github.com/gcollet/MstatX). Gene trees were reconstructed using RAxML 8.2.4 (Stamatakis, 2014) with 1000 rapid bootstrap replicates (BS) and the GTRGAMMA model. Summary statistics were generated using AMAS (Borowiec, 2016) and plotted using R 4.3.2 (R Core Team, 2023). The multispecies coalescent model implemented in ASTRAL‐III (Zhang et al., 2018) was used to construct species trees with default settings. For usage in subsequent analysis (PhyParts), an additional species tree was constructed with ASTRAL‐III from gene trees with <50% BS collapsed branches into a polytomy created in TreeCollapserCL 4 (Hodcroft, 2016). Additionally, alignments of all low‐copy genes were concatenated and analyzed by taking the supermatrix approach using FastTree 2 (Price et al., 2010). An overview of the reads obtained for all taxa involved is given in Table S1.

### Alternative species trees

To achieve better insight into the reliability of the ASTRAL species tree, we also provide a Bayesian multispecies coalescent framework for species tree estimation from multilocus sequence data via BEAST2 version 2.6.7 (Bouckaert et al., 2019) and specifically starBEAST2 (*BEAST2; Ogilvie et al., 2017). Because *BEAST2 works at an acceptable speed with a limited number of genes, we implemented several ways to reduce the number of genes (nuclear loci) based on gene tree comparison. In the first approach, Robinson–Foulds distances (Robinson & Foulds, 1981) of all gene trees were calculated to sort them according to their similarity to the ASTRAL species tree. Subsequently, we chose 40 nuclear loci that provide trees with the highest similarity rate. In the second approach, we implemented the SortaDate package (Smith et al., 2018) involving several steps of gene tree sorting according to bipartition concordance with the species tree, root‐to‐tip variance, and tree length. After applying this approach, we used 25 nuclear loci based on the best score in their gene tree comparison. We reduced the number of final genes (in comparison to RF approach) for species tree reconstruction because of the inability to find convergence in *BEAST2 with a higher number of genes. As the last way to select genes that best reflect the ASTRAL species tree, we implemented a ‘monophyly’ approach for particular gene trees in selected control nodes. We applied two ways for defining control nodes and subsequent choices of gene trees that fulfill the criteria depicted in Figure S5. In the first choice, all 44 nuclear loci providing gene trees that were monophyletic in seven control nodes were selected. In the second choice, all 28 nuclear loci providing gene trees that were monophyletic in four particular control nodes and at least in 6 out of 9 other control nodes were selected. The gene (exons) assemblies selected in each of the four above‐described approaches were analyzed via *BEAST2 using multiple sequence alignments under the GTR + G + I model, with a strict clock (clock rate = 0.001) and without dating. Two independent MCMC analyses were carried out by running them for 250 million generations, sampling every 5000 generations. Resulting trees and logs were mixed using LogCombiner version 2.6.7 (BEAST2 subprogram). Effective sample sizes were inspected in program Tracer version 1.7.2 (Rambaut et al., 2018) and summarizations of outputs with 80% burn‐in were made in TreeAnnotator version 2.6.7 (BEAST2 subprogram). Densitree version 2.6.7 (BEAST2 subprogram) was used to visualize the species trees.

To evaluate the level of concordance among gene trees (with a maximum of 30% of missing data and with collapsed nodes with <50% support) against the ASTRAL species tree, we use the program PhyParts (https://bitbucket.org/blackrim/phyparts; Smith et al., 2015). To root the tree, we use the Figtree program (Rambaut, 2018) and the genus *Andinia* as an outgroup. The output obtained with PhyParts was visualized by plotting pie charts on the species trees with the Python script PhyPartsPieCharts (https://github.com/mossmatters/MJPythonNote‐books) using the ETE3 toolkit (Huerta‐Cepas et al., 2016).

### Testing alternative topology scenarios

In addition to the effect of alternative gene selection on the reconstruction of the species tree, we also used a test of the variability of topological support as a function of changes in the representatives of predefined clades. This test consists of selecting individual representatives of defined clades from the species tree (usually at the genus level), pruning all 630 gene trees to these representatives, and recalculating the topological support for all applicable or desired scenarios based on the pruned gene trees. To this end, we have written a script that combines customization steps on how to proceed with random or targeted selection of representatives, computes all necessary outputs, and finally visualizes alternative topologies at the genus level ranked based on decreasing topology support. The computational pipeline is mostly written in R, but the part for computing topology support involving the PhyPart approach is written in bash (Data S1 and S2). Briefly, the script consists of a preparation step (defining the path to the species and gene trees and setting the nodes/clades whose support will be tested), a simplification step (setting how many times a random representative will be selected for each node, and a loop that does this), an alternative scenario step (this step simplifies the number of all possible alternative topologies to a subset of those, that (i) exist in the gene trees and (ii) provide a majority consensus tree with the desired number of nodes), the PhyParts step (computes the PhyPart support for all alternative topologies), and finally the node support step (summarizes the support for each node/clade in all runs of representative selection).

### cpDNA tree reconstruction

Trimmed and deduplicated HybSeq reads (see above) were also used for plastome tree reconstruction. The reads were mapped against plastome reference of *Dryadella lilliputiana* (GenBank, MW375126) using Geneious Prime 2022.2.2 (www.geneious.com) and its own implemented option ‘Map to Reference’. Before the read mapping, one of the two inverted repeats of the plastome reference was removed. The consensus sequence was called under default options (60% highest quality threshold with >50% threshold for consensus variant calling) and minimum read depth of 5. Unmapped regions of the plastome were treated as gaps (−). The alignment was built using MAFFT and edited via R package *ape* and its function del.colgapsonly with the threshold 0.05. This removes all sites with a gap marker for more than 5% of individuals across all positions in the alignment. The final alignment was used for tree reconstruction using RAxML‐NG with GTR + G model.

### Temporal scaling of the species tree

We used the RelTime method (Kumar & Hedges, 2016), a fast‐dating and high‐performance algorithm that is implemented in MEGA X (Mello, 2018). We used a species tree rooted with representatives of two genera outside of target clades (*Stelis* and *Pleurothallis*), ASTRAL species tree as a starting tree and concatenated alignment of full sequences of 44 nuclear loci (selected in our first choice in ‘monophyly’ approach) as source data for branch length estimation. The GUI‐based version of RelTime in MEGA X was utilized, using the RelTime‐ML option for timetree estimation and GTR with four discrete gamma rate categories as a model for the sequence alignment. As the time constraint, we used prior information on divergence of genus *Andreettaea* from the rest of clade D genera (i.e., all other genera with exception of *Andinia*), specifically 9 ± 1.5 Ma (adopted from Chumová et al., 2021).

### Flow cytometry (FCM)

Data on genome size, GC content, and endoreplication mode (partial vs. conventional endoreplication) were estimated via flow cytometric analyses of all samples included in the tree according to the best practices (Sliwinska et al., 2022) and methodology described in detail by Trávníček et al. (2015, 2019). Standards used in genome size estimation for each species are listed in Table S2. The assessment of the type of endoreplication is based on the quantification of the amount of DNA that has undergone endoreplication in the course of cell differentiation. Whereas plants with 100% replicated DNA (endoreplicated nuclei of subsequent sizes always differ by two‐fold) are assigned to the conventional type of endoreplication, plants with less than 100% replicated DNA (endoreplicated nuclei of subsequent sizes always differ by less than two‐fold) are assigned to partial endoreplication. The arbitrary threshold for assignment was set to 95% of replicated DNA because of possible measurement errors. The endoreplicated part of the genome (P) was calculated as a portion of the whole genome and expressed as a ratio (0–1) or as a genome size.

### FCM traits in spatial analysis

A spatial analysis to evaluate trends in FCM traits across the spectrum of species within the target clade was conducted in a pseudo‐occurrence manner. Members of the Pleurothallidinae suffer from the absence of georeferenced localities in the required certainty and taxonomic clarity. Most occurrence data are available in the form of vague descriptions including only country of origin and approximate elevation (e.g., Icones Pleurothallidinarum). Therefore, these data were used to reconstruct pseudo‐range boundaries for each of the 85 taxa for which at least this information was available (six species were excluded because they are described without any distribution data). For this purpose, World Geographic System for Recording Plant Distribution (according to Biodiversity Information Standards—TDWG) data at its internal precision level 3 were used because they are available for plant species (including all orchids) listed in the World Checklist of Vascular Plants (POWO, 2023; available online and continuously updated at https://powo.science.kew.org/) and are accessible through the Build Checklist function. Elevational spans of taxa (minimum and maximum altitude for growth) were obtained from various literature sources containing the original taxon descriptions. Data for all taxa with known distribution are listed in Table S2. Both datasets were used in the reconstruction of taxa distribution ranges based on a raster digital elevation model of the Earth's surface (GMTED2010 global elevation data in the resolution of 30 arc sec ~1 km) and its processing using the *terra* R package (Hijmans et al., 2022). Subsequently, the pseudo‐occurrence ranges of the species were converted to a presence/absence grid that was constructed over the entire range of all 85 species by dividing them into 0.5 × 0.5 arc degree grid cells (i.e., grid cells ~55.7 × 55.7 km). In this way 5472 grid cells across the land surface within the target distribution area were defined. FCM traits (genome size and GC content) estimated for each taxon were projected to grid cells as a weighted mean of the trait for all taxa weighted by their occupied area within each grid cell. To look at spatial distribution of FCM traits in an ecological context, the ecoregions defined in the area (adopted from Omernik & Griffith, 2014 and Griffith et al., 1998) were mapped over the grid to visualize possible trends. In addition, mean values for 19 bioclimatic variables (adopted from Karger et al., 2017) were extracted for each grid cell and correlated with FCM traits. All spatial and statistical analyses were conducted in R and suitable packages (R Core Team, 2023). Presence of the particular species was counted as 1 if its virtually occupied area exceeded 5% of the grid cell area, otherwise the cell grid was counted as unoccupied (i.e., 0).

### Evolution of FCM traits

Dated ASTRAL species tree based on nuclear low‐copy genes was used for visualization of FCM trait evolution via R package *phytools* (Revell, 2012) and implemented function contMap. Subsequently, the script for determining significant changes in a trait along phylogeny (according to Šmarda et al., 2014) was used. Correlation of FCM traits was conducted in phylogenetic independent contrast, that is, via trait transformation with pic function in R package *ape* (Paradis & Schliep, 2019). Estimation of the phylogenetic signal of all traits and their relationships was calculated using the phylogenetic generalized least squares (PGLS) in the *caper* R package (Orme et al., 2018). To achieve insight into correlation of genomic traits with environmental data in phylogenetic context, bioclimatic variables (Karger et al., 2017) were extracted for taxa in phylogeny. However, the PGLS analysis works with single values per tip in phylogeny, so the bioclim variables were expressed in several ways in order to underpin their importance across their range. All bioclim variables and altitude for all raster cells in the pseudo‐occurrence area were extracted and calculated their percentiles (at the 10, 25, 50, 75, and 90% value), mean, and standard deviation. Subsequently, the regression analysis of these data with FCM traits was performed via PGLS. To analyze data with uncertainty the sensiPhy package and specifically the function intra_phylm was employed (Paterno et al., 2018). At first, mean values ± standard deviation of environmental (bioclim) and altitude data were used. Considering the highly variable data in respect to pseudo‐occurrence origin, the second way of involving uncertainty was using 50% percentile data ±2.5% or 5% percentiles (i.e., calculation of medians with two levels of uncertainty given by differences of percentiles 47.5–52.5% and 45–55%, respectively). The analyses were replicated 1000 times by setting parameter n.intra.

### Biogeography

In our investigation of the biogeographic history of clade C and D, we used the R package *BioGeoBEARS* version 1.1.2 (Matzke, 2013). *BioGeoBEARS* offers a versatile, likelihood‐based framework designed to model how phylogenetic branches, representing both extant taxa and their ancestral lineages, have undergone transitions among discrete biomes over time. Utilizing both the phylogenomic information and distribution data, we applied three distinct models that encompass a range of cladogenetic and anagenetic events. These models included likelihood implementations of Dispersal‐Vicariance Analysis (DIVALIKE; Ronquist, 1997), Dispersal‐Extinction‐Cladogenesis (DEC; Ree and Smith, 2008), and BayArea (BAYAREALIKE; Landis et al., 2013), all integrated within the *BioGeoBEARS* framework (Matzke, 2013).

We divided the occurrence area of clades C and D into seven discrete ecoregions and assigned each species to one or more of them based on their occurrence records. Ecoregions classifications were adopted from Omernik & Griffith (2014; North America) and Griffith et al. (1998; Central and South America). The maximum number of areas was set to seven. AICc was used for *a posteriori* test of models. To assess the contributions of various biogeographic events to the evolution of the Clade D (Dupin et al., 2017), we utilized biogeographic stochastic mapping also through the R package *BioGeoBEARS* (Matzke, 2013). The models tested incorporated different biogeographic events, including speciation within the same area (sympatric speciation), speciation within a subset of the area (parapatric speciation), vicariance, founder events, range expansion, and range contraction (Dupin et al., 2017). The analysis employed a nontime‐stratified regime with 50 stochastic replicate maps for biogeographic stochastic mapping.

## CONFLICT OF INTEREST

The authors declare no conflicts of interest.

## Supporting information


**Data S1.** An annotated custom script written in R to explore plausible evolutionary scenarios provided by all available gene trees.

**Data S2.** A simple BASH script to calculate PhyParts for the desired key tree clade representatives and all evolutionary scenarios investigated.

**Figure S1.** starBEAST species tree estimation from 40 sequence datasets (nuclear loci) selected based on the highest similarity rate using Robinson‐Foulds distances of gene trees from the ASTRAL species tree.
**Figure S2.** starBEAST species tree estimation from 25 sequence datasets (nuclear loci) selected based on the highest similarity rate using the SortaDate approach.
**Figure S3.** starBEAST species tree estimation from 44 sequence datasets (nuclear loci) selected based on gene tree monophyly in particular seven nodes.
**Figure S4.** starBEAST species tree estimation from 28 sequence datasets (nuclear loci) selected based on gene tree monophyly in four particular nodes and in at least six out of nine other particular nodes.
**Figure S5.** Two ways of defining ‘monophyly’ control nodes and subsequent choice of gene trees that fulfill particular criteria.
**Figure S6.** Co‐phylogenetic plot with the ASTRAL species tree on the left side and the RAxML cpDNA tree on the right side.
**Figure S7.** The top twelve alternative topologies revealed by altering the genus/clade representatives from the original ASTRAL species tree.
**Figure S8.** Analyses of *Specklinia guanacastensis* by flow cytometry with two different standards and two fluorescent dyes.
**Figure S9.** Difference in GC content of nuclei that underwent different numbers of runs of endoreplication.
**Figure S10.** Evolution of the genome size of the replicated part of the genome visualized by mapping on the ASTRAL species tree.
**Figure S11.** Evolution of GC content visualized by mapping on the ASTRAL species tree.
**Figure S12.** Relationship between GC content and genome size.
**Figure S13.** Species distribution densities across the target area as a number of species per grid cell.
**Figure S14.** Relationship between genome size and two bioclimatic variables that have been shown to be most important in explaining genome size variability using PGLS.

**Figure S15.** Bayes factor plot for the BAMM analysis, showing that the posterior probability for no shifts in speciation rates highly surpass the prior distribution.
**Figure S16.** Phylogeny of clade D indicating no diversification in rate shifts.
**Figure S17.** Rate‐through time density plot for clade D, depicting a constant speciation rate.
**Figure S18.** Diversification analyses plots derived from BiSSE, when computing partial and complete endoreplication with the rate of speciation (a) and extinction (b).
**Table S12.** Summary of BiSSE model fitting.
**Table S13.** Summary of HiSSE model fitting.
**Table S14.** Summary of QuaSSE model fitting.

**Table S1.** List of specimens included in the study, supplemented by HybSeq statistics.
**Table S2.** Summary of included representatives of the subtribe Pleurothallidinae with data obtained by flow cytometry and species distribution data.
**Table S3.** The effect of removing individual species on tree topology support.
**Table S4.** PGLS results based on comparison of FCM traits and environmental data.
**Table S5.** Results of different models of BioGeoBEARS.
**Table S6.** Summary of biogeographic stochastic mapping results for the clade D and the best‐fit model (DEC + J).
**Table S7.** Origin range probability of the main clades of the clade D tree.
**Table S8.** All dispersal events across operational areas.
**Table S9.** Founder events across operational areas.
**Table S10.** Range expansion events across operational areas.
**Table S11.** Results of Chi‐squared test for sampling strategy across all genera in targeted clades.

## Data Availability

Sequence data are available in a public repository deposited in GenBank (https://www.ncbi.nlm.nih.gov/sra) with the primary accession codes PRJNA604559 and PRJNA1172921. All computational pipelines and respective input data are available at the GitHub repository https://github.com/PT‐ibot/cladeD_repo.
