# Supplementary material for: Integrating genome‐wide traits and multi‐loci phylogeny to investigate orchid evolution—A case study on Pleurothallidinae
Source: Plant J. 2025 Jun 20;122(6):e70281. doi: 10.1111/tpj.70281 (PMC12179579; doi:10.1111/tpj.70281)
Supplement: Supplementary file 3 — Figure S1. starBEAST species tree estimation from 40 sequence datasets (nuclear loci) selected based on the highest similarity rate using Robinson‐Foulds distances of gene trees from the ASTRAL species tree. Figure S2. starBEAST species tree estimation from 25 sequence datasets (nuclear loci) selected based on the highest similarity rate using the SortaDate approach. Figure S3. starBEAST species tree estimation from 44 sequence datasets (nuclear loci) selected based on gene tree monophyly in particular seven nodes. Figure S4. starBEAST species tree estimation from 28 sequence datasets (nuclear loci) selected based on gene tree monophyly in four particular nodes and in at least six out of nine other particular nodes. Figure S5. Two ways of defining ‘monophyly’ control nodes and subsequent choice of gene trees that fulfill particular criteria. Figure S6. Co‐phylogenetic plot with the ASTRAL species tree on the left side and the RAxML cpDNA tree on the right side. Figure S7. The top twelve alternative topologies revealed by altering the genus/clade representatives from the original ASTRAL species tree. Figure S8. Analyses of Specklinia guanacastensis by flow cytometry with two different standards and two fluorescent dyes. Figure S9. Difference in GC content of nuclei that underwent different numbers of runs of endoreplication. Figure S10. Evolution of the genome size of the replicated part of the genome visualized by mapping on the ASTRAL species tree. Figure S11. Evolution of GC content visualized by mapping on the ASTRAL species tree. Figure S12. Relationship between GC content and genome size. Figure S13. Species distribution densities across the target area as a number of species per grid cell. Figure S14. Relationship between genome size and two bioclimatic variables that have been shown to be most important in explaining genome size variability using PGLS. [file TPJ-122-0-s004.pdf]

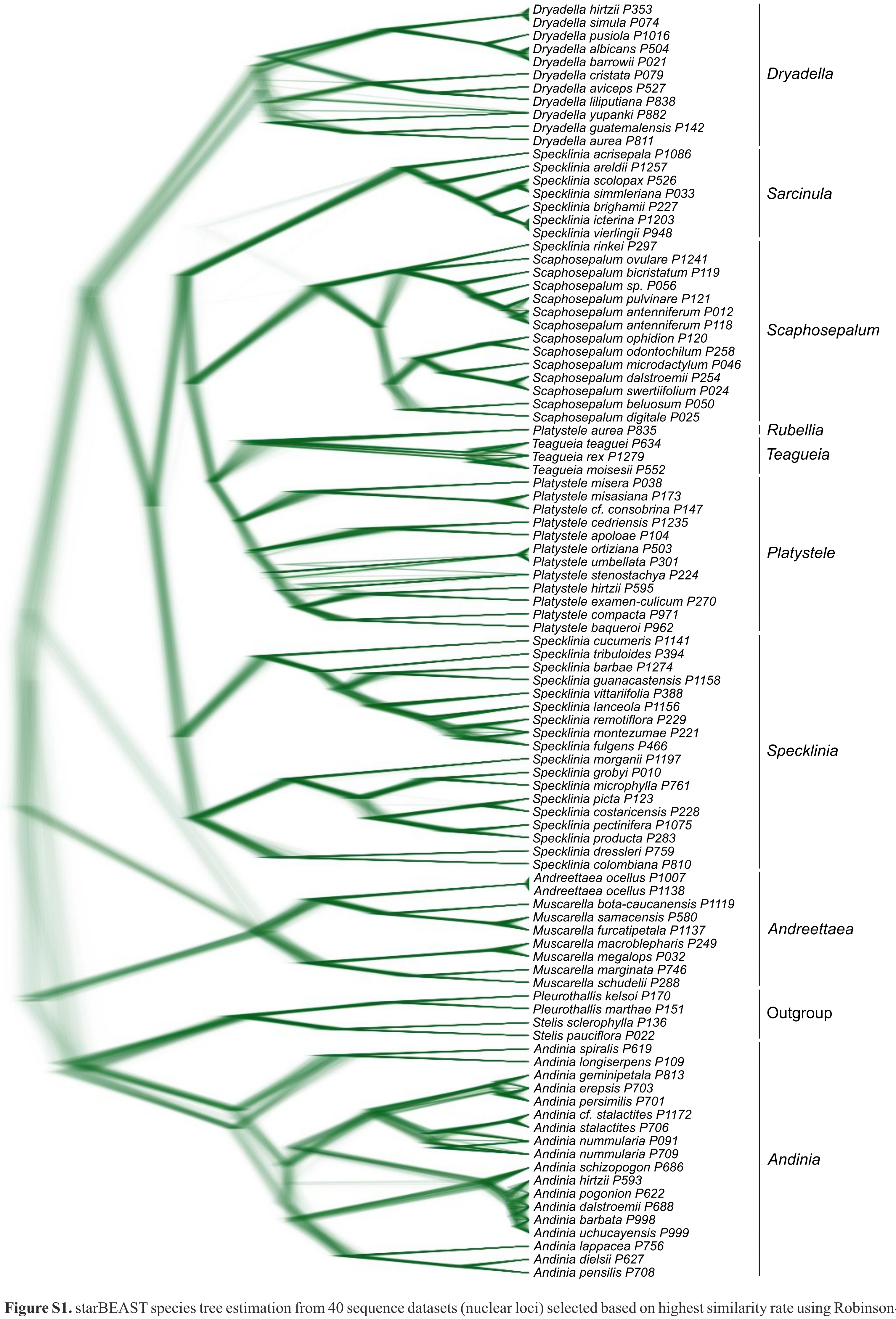

Figure S1. starBEAST species tree estimation from 40 sequence datasets (nuclear loci) selected based on highest similarity rate using Robinson-Foulds distances of gene trees from ASTRAL species tree. For the sake of visual simplicity, only the last 20% of gathered output trees are shown via the Densitree program. Dataset is supplemented with four outgroup taxa (from the sister clade comprising three genera – *Pabstiella*, *Pleurothalis*, and *Stelis*).

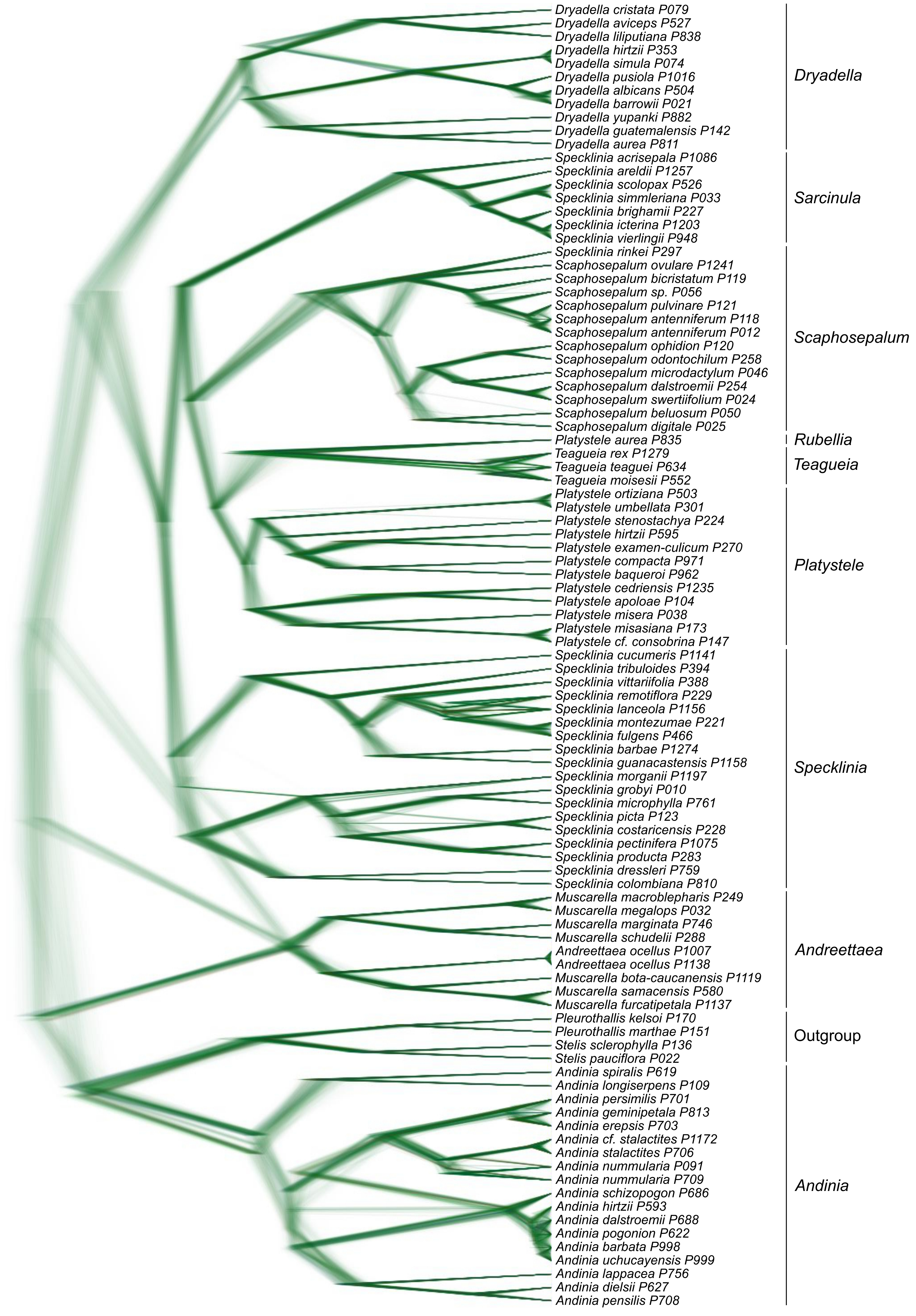

Figure S2. starBEAST species tree estimation from 25 sequence datasets (nuclear loci) selected based on highest similarity rate using SortaDate approach (see Experimental Procedures for more details). The ASTRAL species tree was used as a reference tree for loci and corresponding gene trees selection. For the sake of visual simplicity, only the last 20% of gathered output trees are shown via the Densitree program. Dataset is supplemented with four outgroup taxa (from the sister clade comprising three genera – *Pabstiella*, *Pleurothalis*, and *Stelis*).

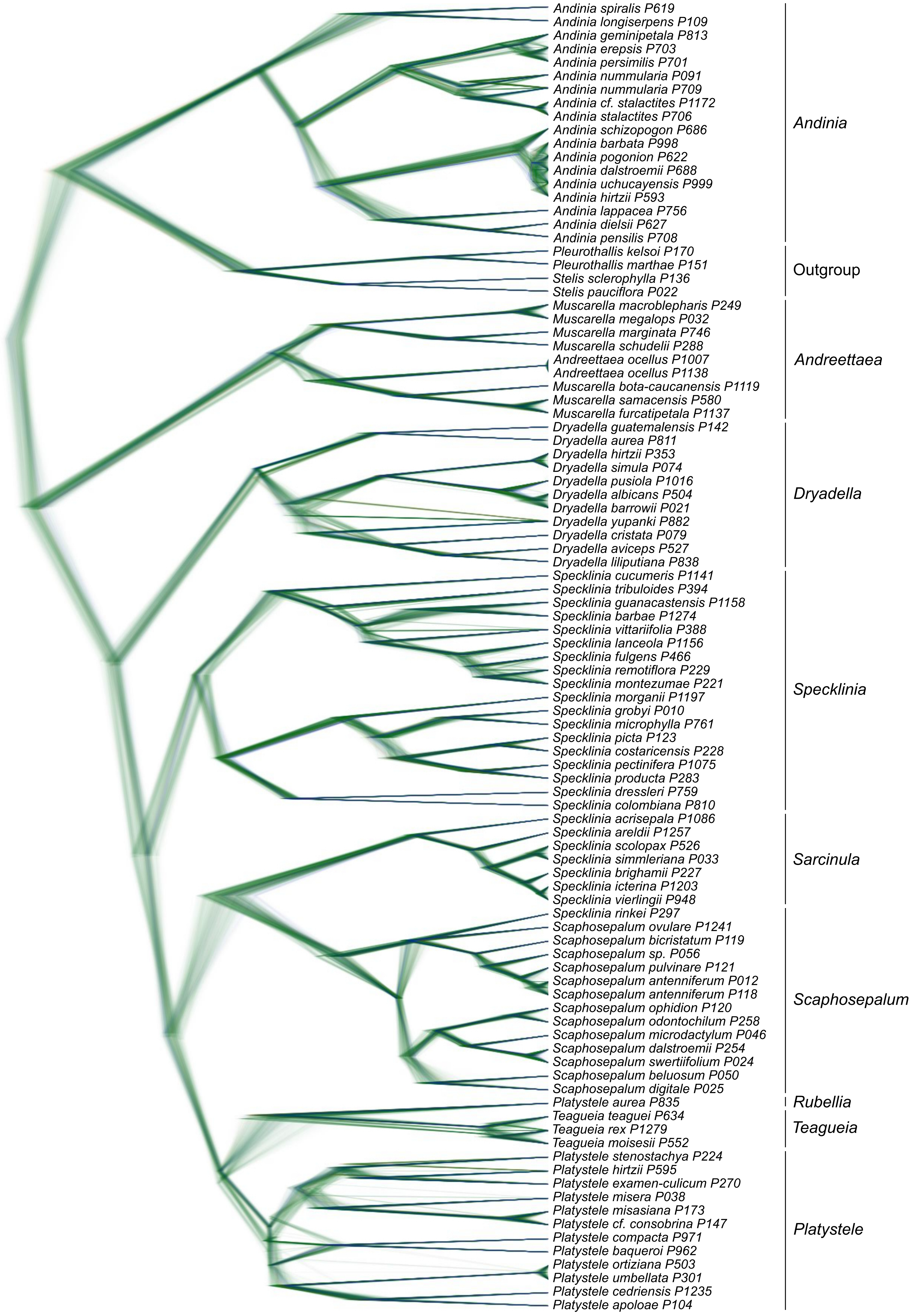

Figure S3. starBEAST species tree estimation from 44 sequence datasets (nuclear loci) selected based on gene tree monophyly in particular seven nodes (see Experimental Procedures section and Figure S5 for details). For the sake of visual simplicity, only the last 20% of gathered output trees are shown via the Densitree program. Dataset is supplemented with four outgroup taxa (from the sister clade comprising three genera – *Pabstiella*, *Pleurothalis*, and *Stelis*).

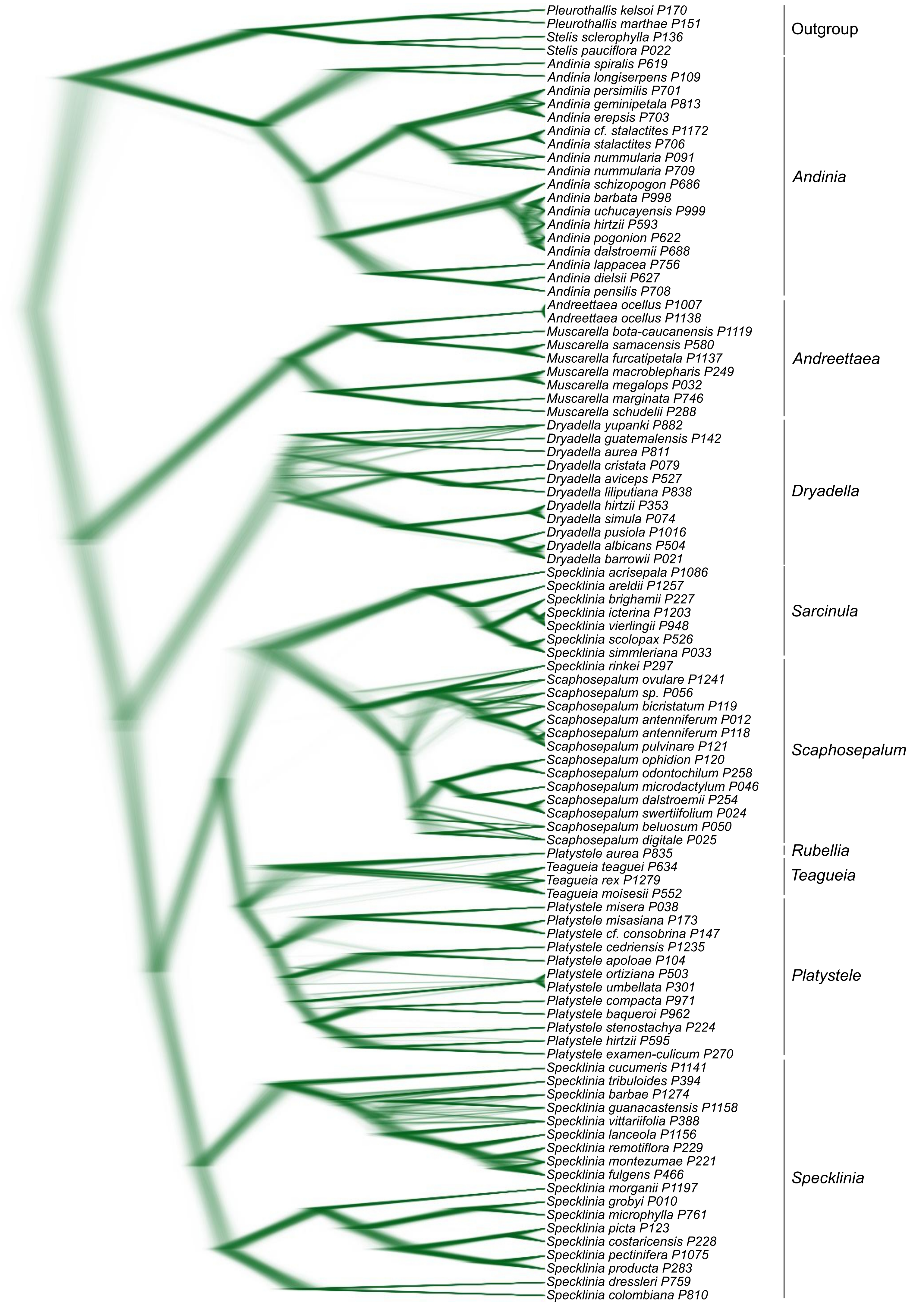

Figure S4. starBEAST species tree estimation from 28 sequence datasets (nuclear loci) selected based on gene tree monophyly in four particular nodes and at least in six out of other nine particular nodes (see Experimental Procedures section and Figure S5 for details). For the sake of visual simplicity, only the last 20% of gathered output trees are shown via the Densitree program. Dataset is supplemented with four outgroup taxa (from the sister clade comprising three genera – *Pabstiella*, *Pleurothalis*, and *Stelis*).

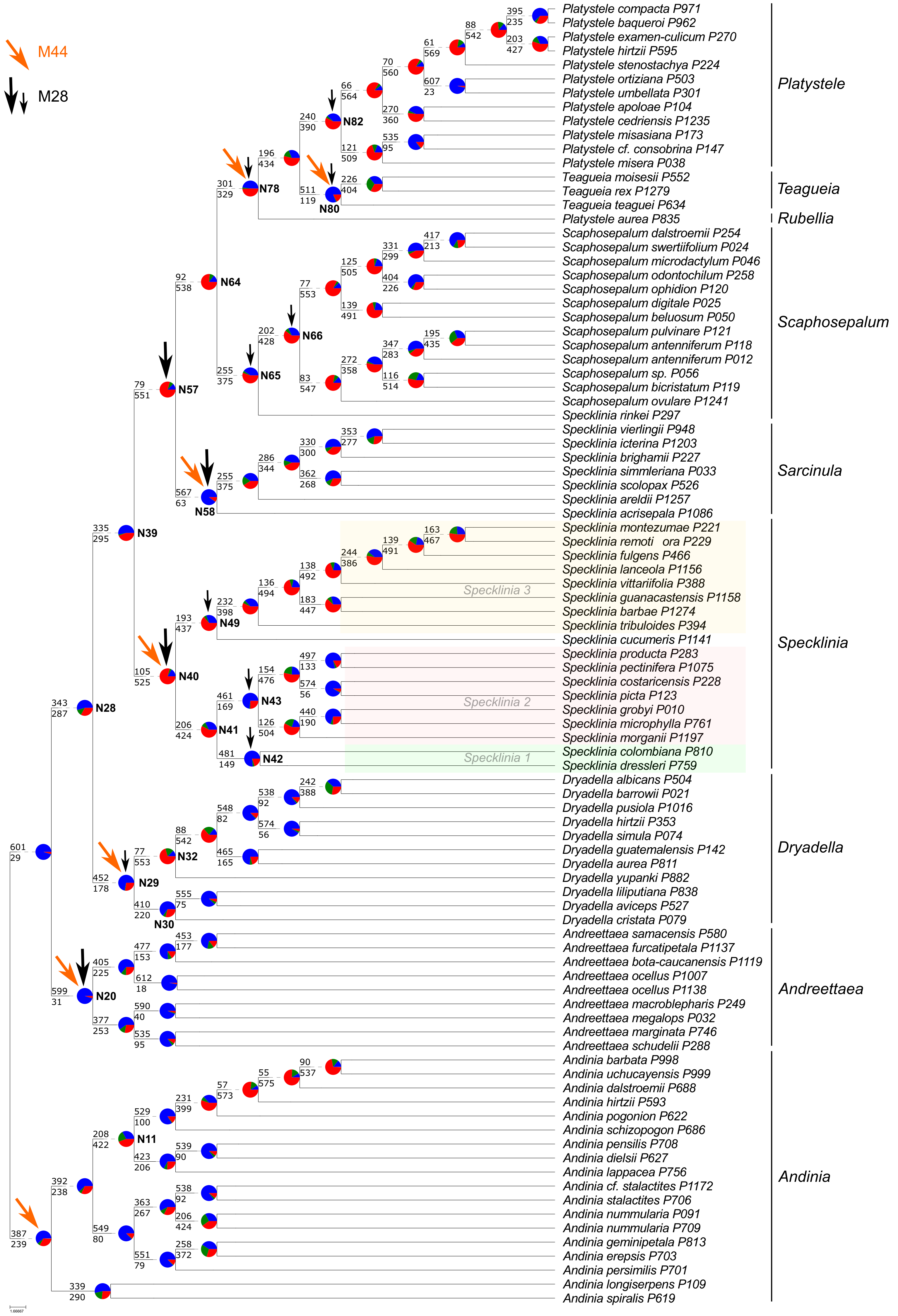

Figure S5. Two ways for defining "monophyly" control nodes and subsequent choice of gene trees that fulfill the following criteria. In the first choice (M44), all 44 nuclear loci providing gene trees that were monophyletic in seven control nodes were selected (marked by orange arrows). In the second choice (M28), all 28 nuclear loci providing gene trees that were monophyletic in four particular control nodes (big black arrow) and at least in 6 out of 9 other control nodes (small black arrow) were selected. ASTRAL species tree built on 630 nuclear gene RAxML trees serves as a source tree. Nodes are supplemented with a PhyPart graphical overview showing the proportion of congruent and incongruent gene tree topologies. Blue color indicates proportion of congruent topology, green color proportion of main alternative topology, and red color proportion of incongruent topology. The numbers above branches indicate the number of gene trees with the same topology, numbers below branches count the number of incongruent gene trees. Node numbers (N##) by selected nodes are mentioned in the paper and/or listed in Table S3.

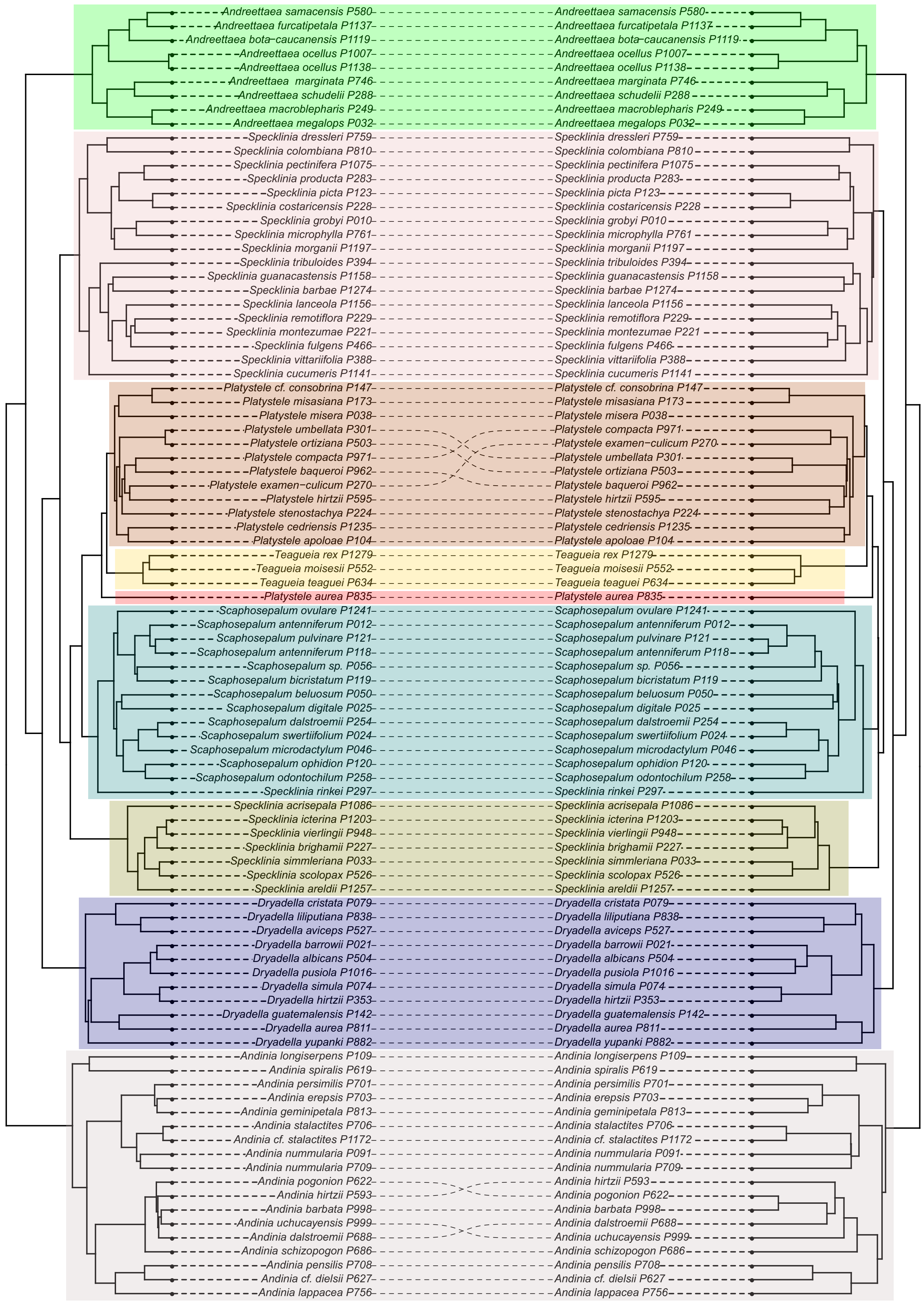

Figure S6. Co-phylogenetic plot with ASTRAL species tree on left side and RAxML cpDNA tree on the right side. ASTRAL tree represents a species tree reconstructed from 630 gene trees based on nuclear low-copy loci. cpDNA tree is based on concatenated 22 290 bp long alignment of reconstructed parts of plastome across all species in phylogeny.

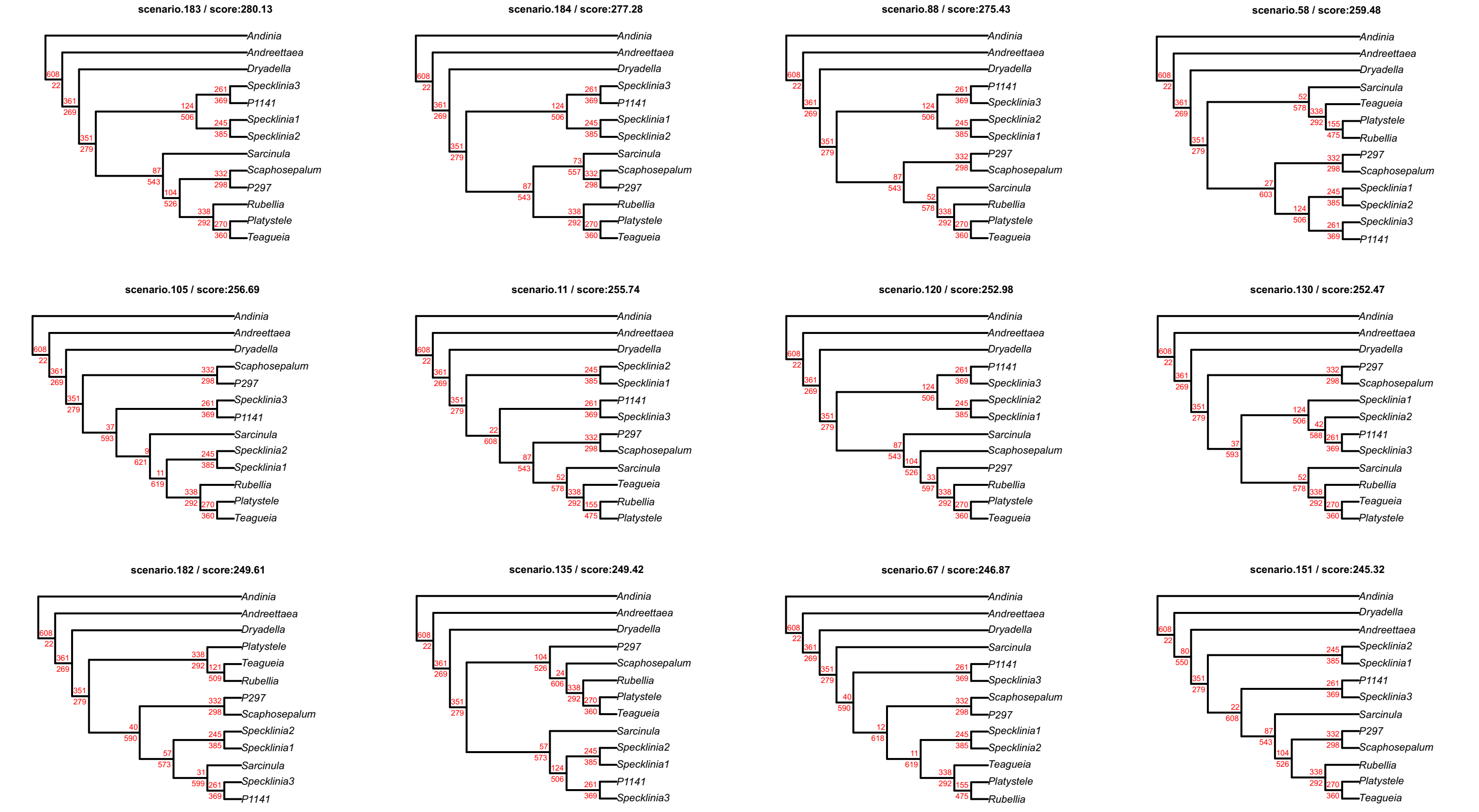

Figure S7. The top twelve alternative topologies revealed by altering the genus/clade representatives from the original ASTRAL species tree (see the Experimental Procedures section for a detailed description). The numbers above the branches indicate the average number of gene trees (out of a total of 630 trees) supporting a given topology, while the numbers below the branches indicate the average number of gene trees exhibiting a different topology. The genus/clade labels mostly correspond to the lineages defined in Figure S5. Due to the separate position of two taxa, *Specklinia rinkei* (labeled as P297) and *Specklinia cucumeris* (labeled as P1141), their relationship to other genera/clades is revealed in all topologies.

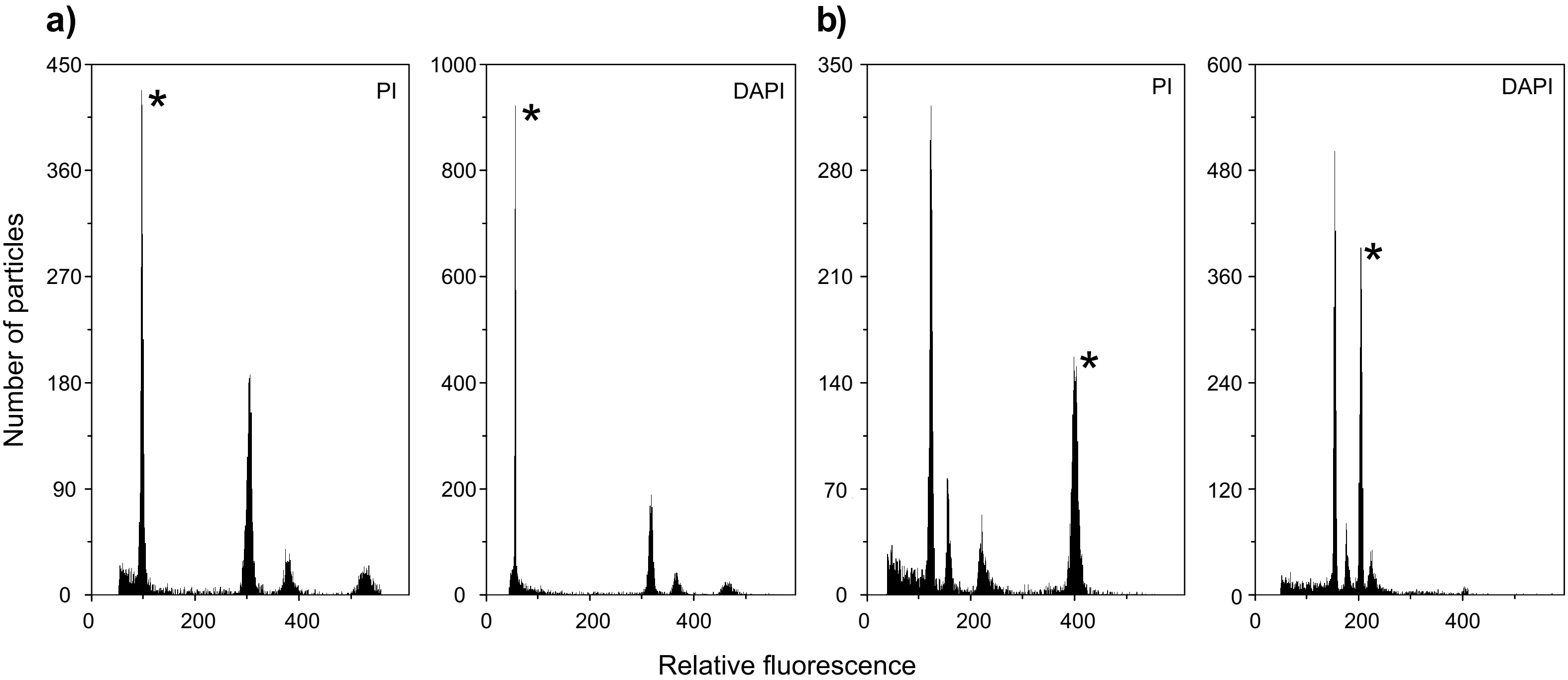

Figure S8. Analyses of *Specklinia guanacastensis* by flow cytometry with two different standards (marked with an asterisk in all histograms) and two fluorescent dyes: intercalating propidium iodide (PI) and AT-selective 4′,6-Diamidino-2-phenylindole dihydrochloride (DAPI). a) Analyses using *Carex acutiformis* (1C = 0.443 pg) as standard and resulting dye factor DF = 1.819. b) The same with *Pisum sativum* (1C = 4.380 pg) as standard and DF = 2.341. Considering the different base composition of the two standards, the resulting GC content of *S. guanacastensis* was estimated to be the same at 22.5%.

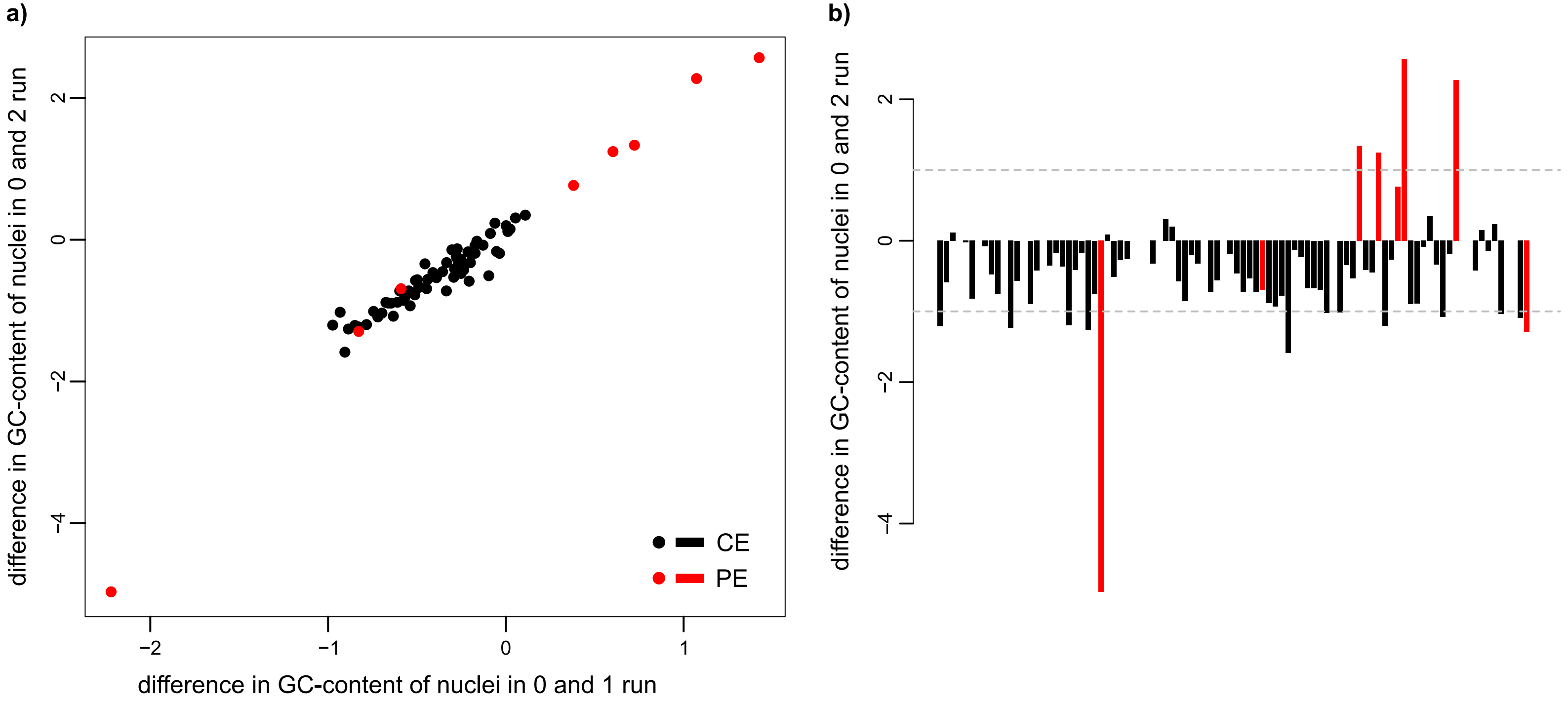

Figure S9. Difference in GC-content of nuclei that underwent different numbers of runs of endoreplication. a) Comparison of GC-content shifts between nuclei with zero and first run of endoreplication (x-axis) and nuclei with zero and second run of endoreplication (y-axis). b) Bar plot showing maximum observed shifts in GC-content for all species with nuclei recorded in zero and second run of endoreplication. Grey dashed lines correspond to shifts in 1% decrease or increase. Species endoreplication type is indicated by color - black for conventional and red for partial endoreplication.

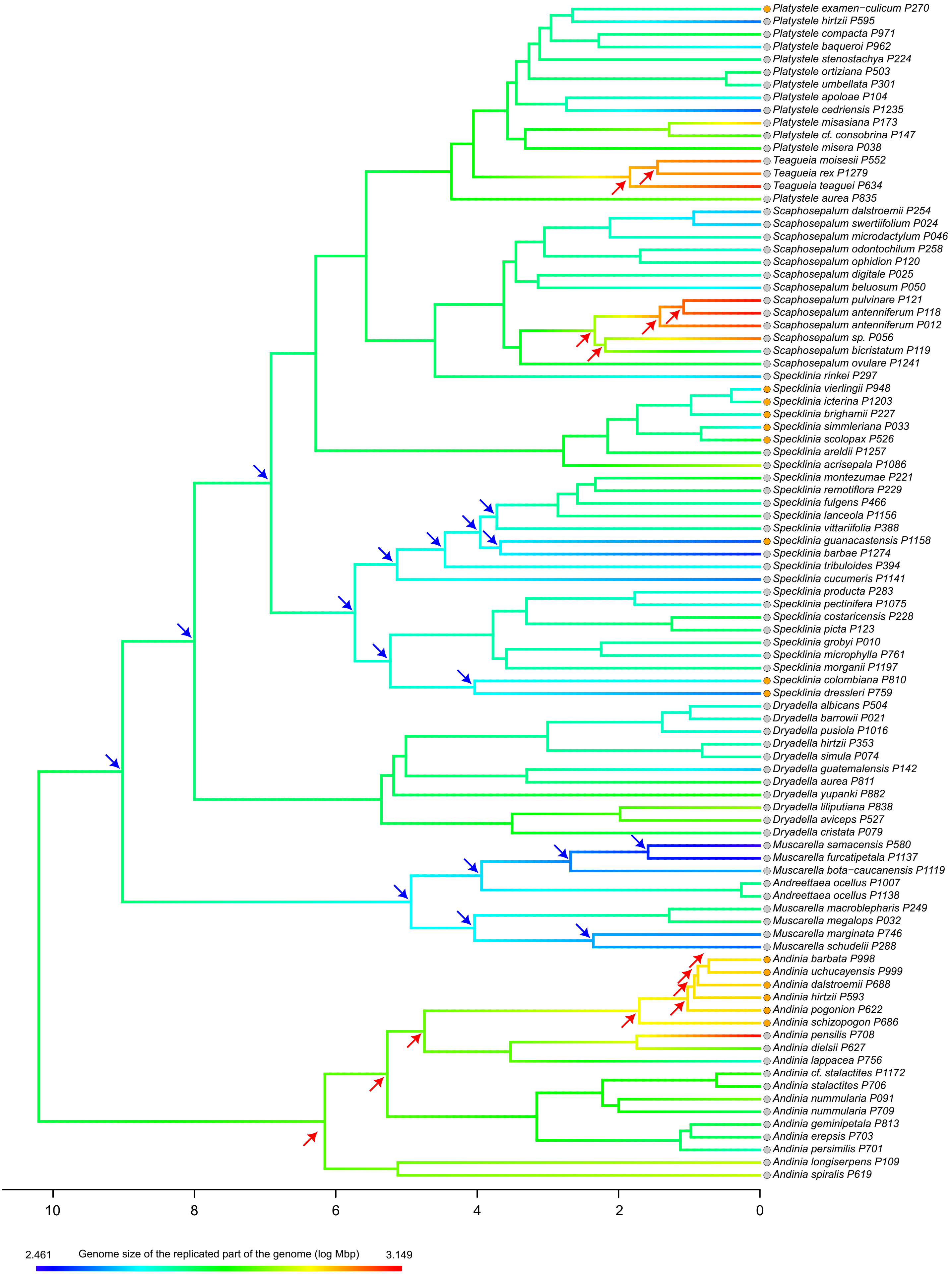

Figure S10. Evolution of the genome size of the replicated part of the genome visualized by mapping on the ASTRAL species tree. The significant changes along the phylogeny are computed according to Šmarda et al. (2014) and depicted by arrows – blue one for decrease, red one for increase. All involved taxa are labeled by colored dots according to type of endoreplication – orange one for partial endoreplication, gray one for conventional endoreplication.

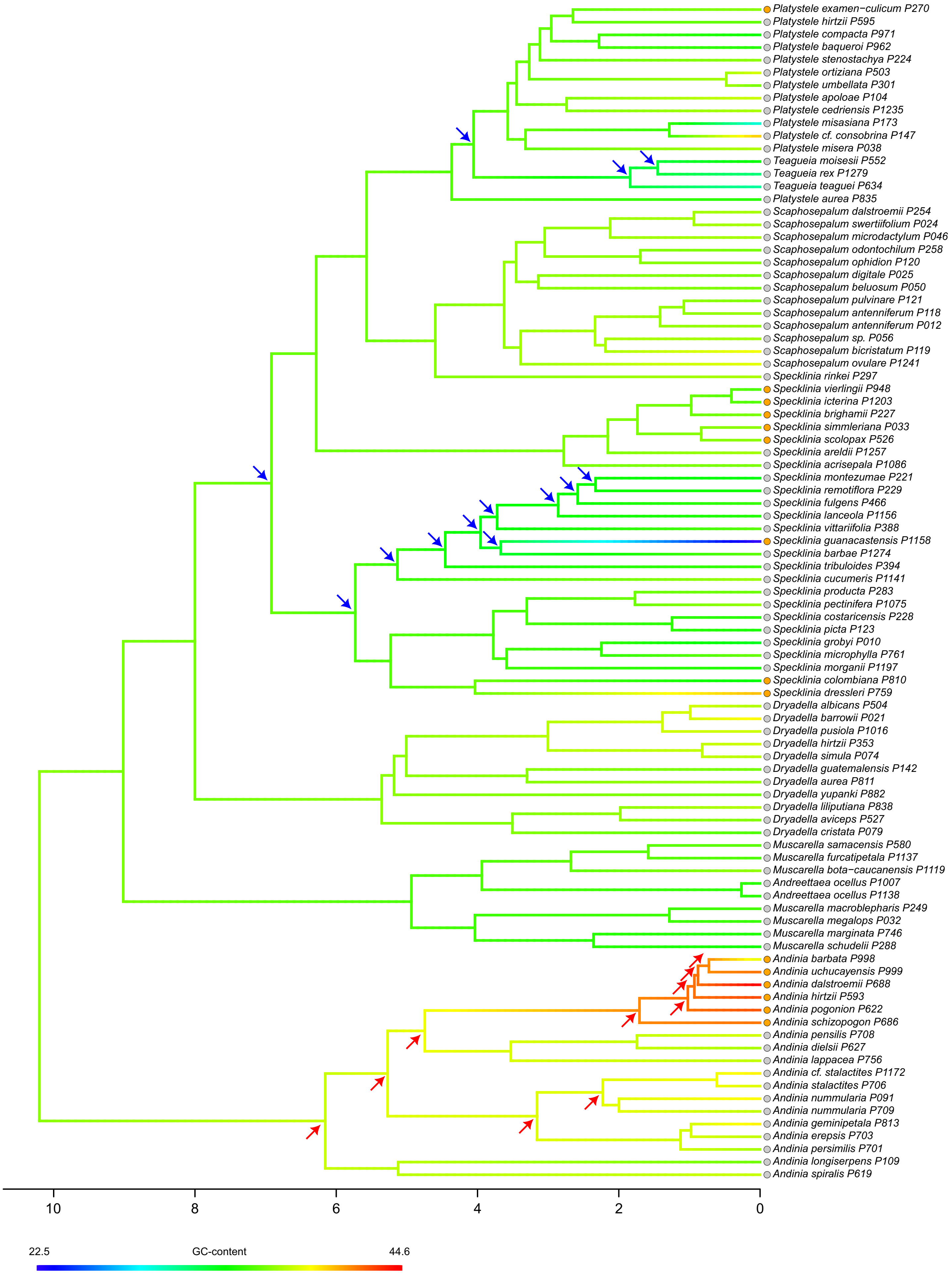

Figure S11. Evolution of GC-content visualized by mapping on the ASTRAL species tree. The significant changes along the phylogeny are computed according to Šmarda et al. (2014) and depicted by arrows – blue one for decrease, red one for increase. All involved taxa are labeled by colored dots according to type of endoreplication - orange one for partial endoreplication, gray one for conventional endoreplication.

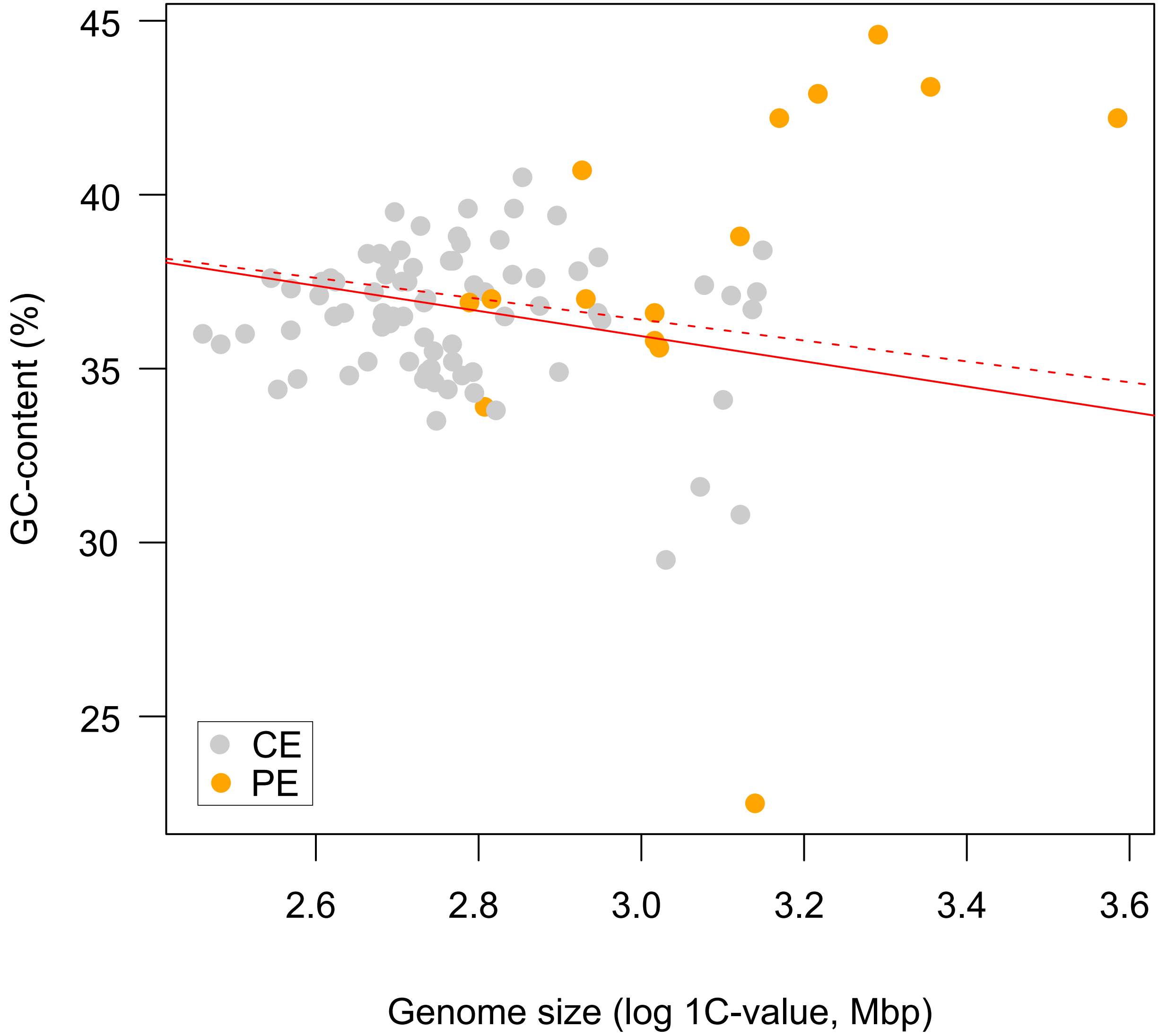

Figure S12. Relationship between GC content and genome size shows marginally insignificant negative correlation (pPGLS = 0.058; dashed red line). When the dataset is reduced to orchids with only conventional endoreplication (78 of 93 accessions), the relationship becomes strongly significant (pPGLS = 0.009; solid red line).

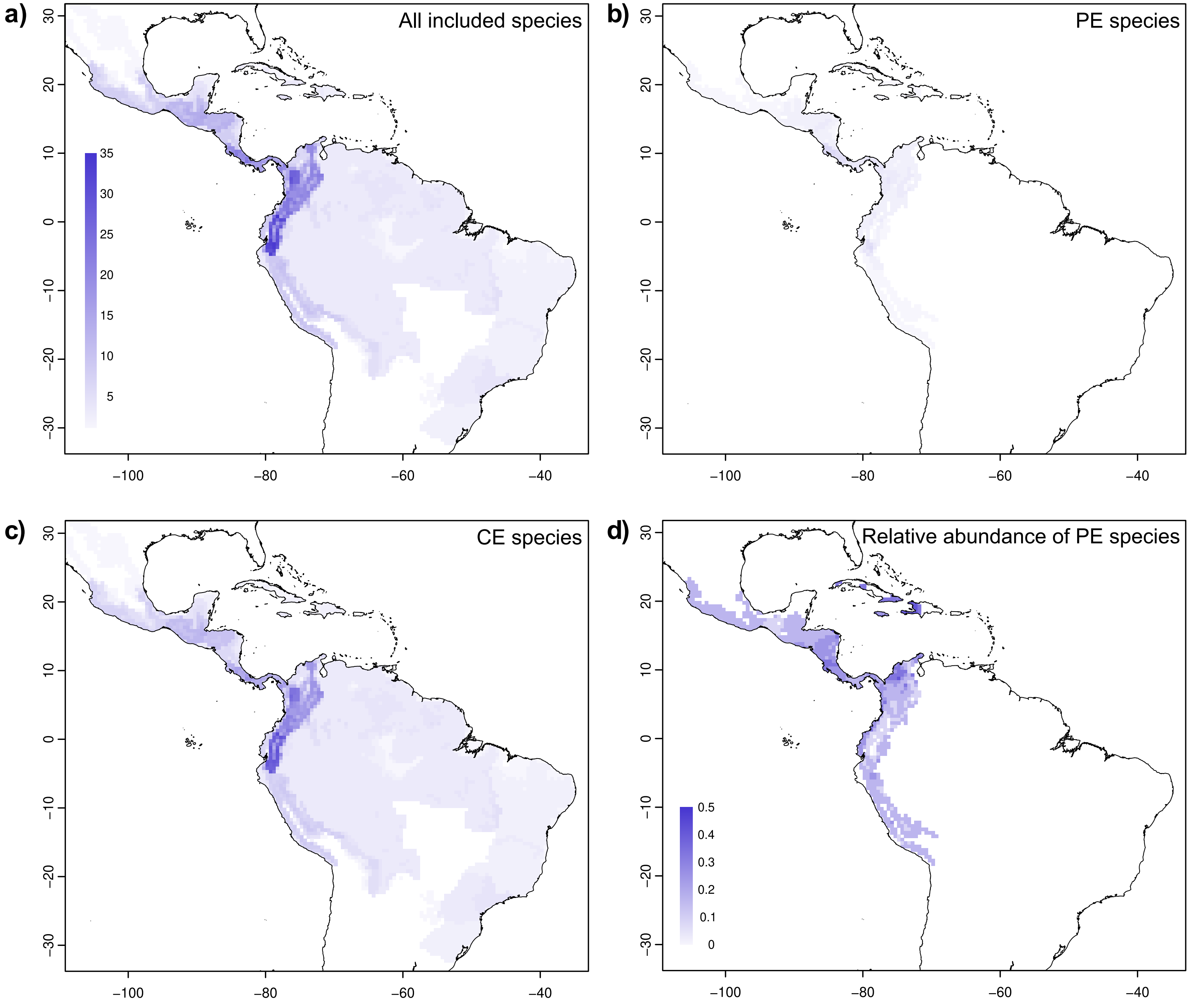

Figure S13. Species distribution densities across the target area as a number of species per grid cell. a) All species included in spatial analysis; b,c) species density for PE and CE taxa, respectively; d) relative abundance of PE species as a fraction of total number of species per grid cell.

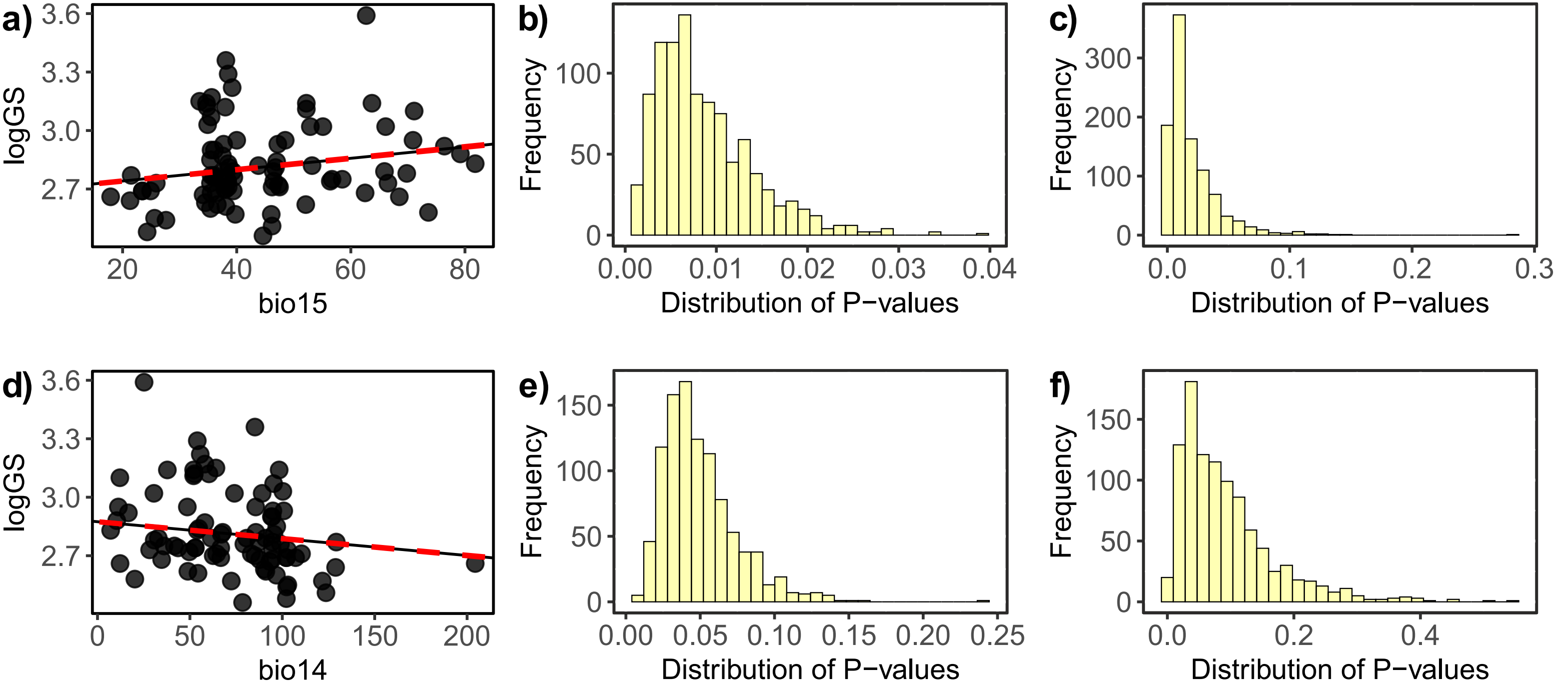

Figure S14. Relationship between genome size and two bioclimatic variables that have been shown to be most important in explaining genome size variability using PGLS. (a–c) Graphical outputs from sensiPhy analysis for seasonality of precipitation (bio15); (d–f) same for precipitation in the driest month (bio14). (a,d) Trends between the explained variable (genome size) and the explanatory variable expressed in median values for all included taxa. (b,c,e,f) Histograms of inferred probabilities in 1000 simulations for median values with allowed variation in the explanatory variable – (b,e) for median ± 2.5%, (c,f) for median ± 5%.
