# Supplementary material for: Integrating genome‐wide traits and multi‐loci phylogeny to investigate orchid evolution—A case study on Pleurothallidinae
Source: Plant J. 2025 Jun 20;122(6):e70281. doi: 10.1111/tpj.70281 (PMC12179579; doi:10.1111/tpj.70281)
Supplement: Supplementary file 4 — Figure S15. Bayes factor plot for the BAMM analysis, showing that the posterior probability for no shifts in speciation rates highly surpass the prior distribution. Figure S16. Phylogeny of clade D indicating no diversification in rate shifts. Figure S17. Rate‐through time density plot for clade D, depicting a constant speciation rate. Figure S18. Diversification analyses plots derived from BiSSE, when computing partial and complete endoreplication with the rate of speciation (a) and extinction (b). Table S12. Summary of BiSSE model fitting. Table S13. Summary of HiSSE model fitting. Table S14. Summary of QuaSSE model fitting. [file TPJ-122-0-s005.pdf]

**Supplementary Material**

**Material and Methods**

*Bayesian Analysis of Macroevolutionary Mixtures (BAMM) analysis*

To assess changes in diversification regimes, encompassing speciation and extinction rates across the phylogeny, we employed BAMM 1.0 along with the BAMMtools R package (Rabosky, 2014). This methodology employs reversible jump Markov chain Monte Carlo (MCMC) to examine models featuring different shift regimes that explain the heterogeneity of evolutionary rates. Our analysis focused on 87 species within clade D, and we addressed the issue of nonrandom incomplete taxon sampling by specifying sampling fractions for each lineage individually. MCMC was executed for 50 million generations, with output saved every 1000th generation. Following the generation of likelihood plots, the initial 10% were excluded as burn-in, and the effective sample size for both likelihood and the number of shifts was computed to evaluate convergence.Identification of significant rate shifts involved sampling from all possible sets of shift configurations, and determining nodes where the posterior probabilities summed up to 0.95.

*SSE analyses*

To test whether variation in diversification rates is related to the genome size, we used a series of state-dependent speciation and extinction (SSE) models. The first one, the Binary state-dependent speciation and extinction model (BiSSE) was implemented in the R package 'diversitree' 0.9–6 (FitzJohn *et al.*, 2009), coding species by partial endoreplication (0) and

complete endoreplication (1). BiSSE aims to explain variations in diversification rates through six parameters: the speciation (λ) and extinction (μ) rates for each state, and the transition rate between states (q). The simplest model, requiring the fewest parameters, was sought by constraining λ, μ, and q to be uniform across both states. Akaike information criterion (AIC) and likelihoods were calculated for each model, and a likelihood ratio test determined whether each constrained model significantly differed from the full (unconstrained) model. Among the constrained models that performed comparably to the unconstrained model, the one with the fewest parameters was selected. Additionally, a BiSSE model was implemented using a Bayesian Markov chain Monte Carlo (MCMC) approach, with a uniform prior probability distribution set for all model parameters.

Looking for other answers that could be related with the variation in diversification rates, we also implement the Hidden-state speciation and extinction model (HiSSE) implemented in the 'hisse' R package  (Beaulieu and O'Meara, 2016). This model allows us to consider the possibility that speciation and/or extinction rates exhibit variability not necessarily in context with the measured trait's specific values (i.e., partial vs. complete endoreplication). Instead, the HiSSE model proposes that diversification rates undergo changes influenced by a hidden character trait. Five models were created and compared: Model 1: BiSSE; Model 2: Character-Independent diversification (CID-1), or a "normal null BiSSE", i.e., constant birth-death model; Model 3: CID-2, no influence of endoreplication traits, but a hidden trait; Model 4: CID-4, no influence of endoreplication traits, but four hidden traits; and Model 5: the Full model, the endoreplication and hidden traits all are influencing the rate shifting.

For each model, we assumed a biased taxon sampling, based on the number of species in the phylogeny and all currently described species of the clade D. We then calculated AIC to

identify the best-fitting models. Following the approach by Caetano *et al.* (2018), we averaged the estimates of diversification parameters across all models according to their AIC weights.

Finally, instead as a discrete trait, we hypothesize that the genome size as a continuously value trait could also be related to the speciation rate variation. Therefore, we implemented the Quantitative-trait speciation and extinction model (QuaSSE) (FitzJohn, 2010), also in the R package 'diversitree' 0.9–6 (FitzJohn *et al.*, 2009). We set four models for this analysis, Model 1: CID-1; Model 2: Speciation varying; Model 3: Extinction varying; and Model 4: Full model, both speciation and extinction varying. As we did for our other SSE analyses, we also compared the models, calculating the AIC to identify the best-fitting models.

**Results**

*BAMM*

The likelihood of the BAMM MCMC reached convergence and the post burn-in effective sample sizes were above 200. The Bayes factor plot (Figure S15) favored models with no shifts. Maximum shift credibility (Figure S16) inferred no shifts in diversification regimes (Figure S17).

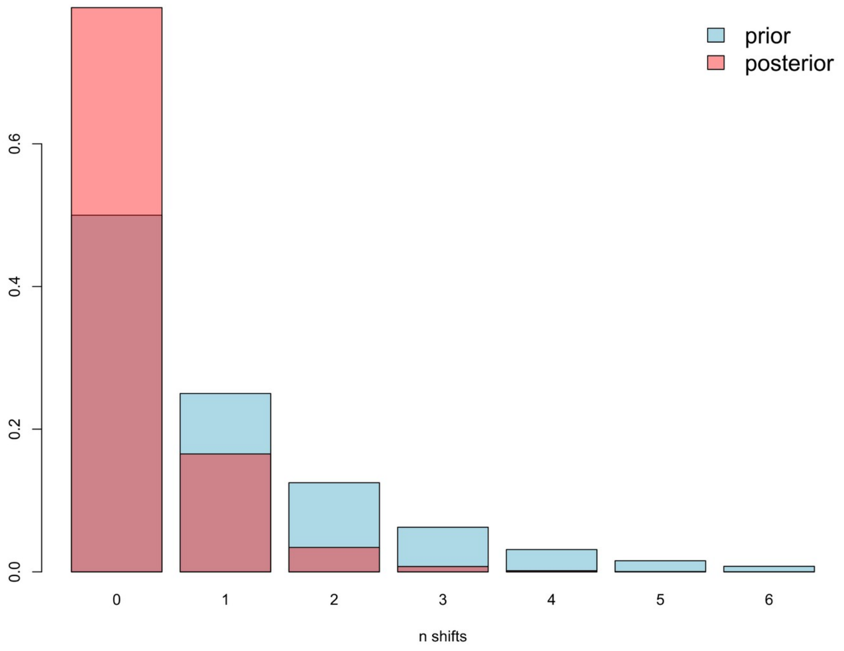


**Figure S15.** Bayes factor plot for the BAMM analysis, showing that the posterior probability for no shifts in speciation rates highly surpass the prior distribution.

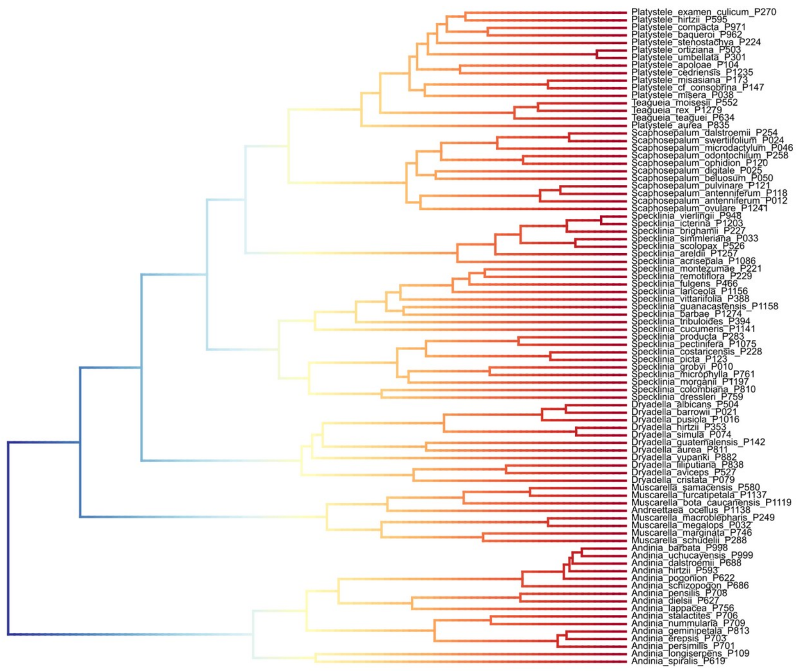


**Figure S16.** Phylogeny of clade D indicating no diversification in rate shifts.

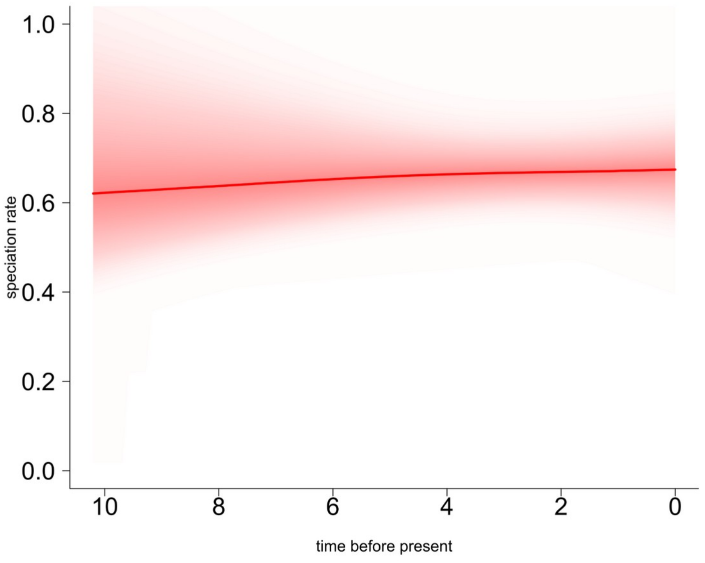


**Figure S17.** Rate-through time density plot for clade D, depicting a constant speciation rate.

*SSE analyses*

All SSE analyses (BiSSE, HiSSE and QuaSSE) showed that the simplest model (i.e., constant evolutionary rate for speciation and extinction) exhibited the highest AIC and likelihood ratio tests when incorporating genome size as a trait (Tables S12-S14). Differences in the diversification rates between lineages with low and high genome sizes could not be accounted for the rate of the variation in speciation regimes. Parameterization of the BiSSE models with Bayesian methods also showed that the relationship between genome size and speciation rates was not significantly higher than the null model (Figure S18).

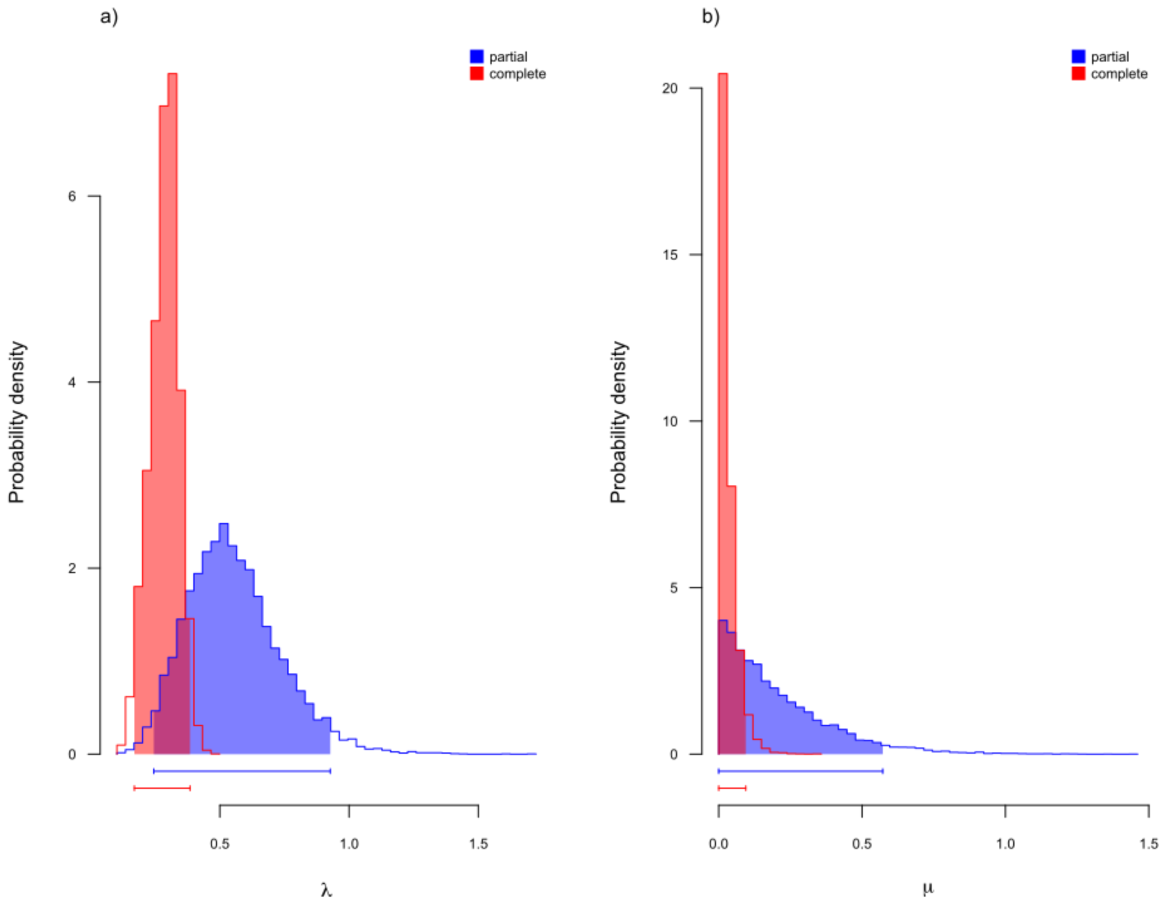


**Figure S18.** Diversification analyses plots derived from BiSSE, when computing partial and complete endoreplication with the rate of speciation (a) and extinction (b).

**Table S12** - Summary of BiSSE model fitting. Df, degrees of freedom; logL, Natural logarithm of the likelihood; AIC, Akaike Information Criterion; ChiSq, Chi-Square; Pr(>|Chi|), p-value for Chi-Square. The best-fitting model is in bold.

| model | *Df* | *logL* | *AIC* | *ChiSq* | *Pr(>\|Chi\|)* |
|---|---|---|---|---|---|
| full | 6 | -207.73699 | 427.473985 | | |
| null | 4 | -208.04215 | **424.084295** | 0.61031052 | 0.73700894 |

**Table S13** - Summary of HiSSE model fitting. logL, Natural logarithm of the likelihood; k, number of parameters; AIC, Akaike Information Criterion. The best-fitting model is in bold.

| model | *logL* | *k* | *AIC* | *Akaike.weight* |
|---|---|---|---|---|
| CID | -208.04213 | 4 | **424.084259** | 0.59720537 |
| BiSSE | -207.73698 | 6 | 427.473956 | 0.10966308 |
| HiSSE CID-2 | -207.7898 | 5 | 425.579598 | 0.28275814 |
| HiSSE CID-4 | -207.09563 | 9 | 432.191261 | 0.01036837 |
| HiSSE full | -206.72681 | 17 | 447.453627 | 5.02-06 |

**Table S14** - Summary of QuaSSE model fitting. Df, degrees of freedom; logL, Natural logarithm of the likelihood; AIC, Akaike Information Criterion; ChiSq, Chi-Square; Pr(>|Chi|), p-value for Chi-Square. The best-fitting model is in bold.

| model | *Df* | *logL* | *AIC* | *ChiSq* | *Pr(>\|Chi\|)* |
|---|---|---|---|---|---|
| minimal | 3 | -61.195002 | **128.390005** | NA | NA |
| variable.lambda | 4 | -60.46581 | 128.931619 | 1.45838567 | 0.22718715 |
| variable.mu | 4 | -61.195234 | 130.390468 | -0.0004631 | 1 |
| full.model | 5 | -60.465819 | 130.931637 | 1.4583679 | 0.48230241 |

**References**

**Beaulieu J. M. and O'Meara B. C.** (2016) Detecting Hidden Diversification Shifts in Models of Trait-Dependent Speciation and Extinction. *Systematic Biology*, **65**, 583–601. https://doi.org/10.1093/sysbio/syw022

**Caetano D. S., O'Meara B. C. and Beaulieu J. M.** (2018) Hidden state models improve state-dependent diversification approaches, including biogeographical models. *Evolution*, **72**, 2308–2324. https://doi.org/10.1111/evo.13602

**Dupin J., Matzke N. J., Särkinen T., Knapp S., Olmstead R. G., Bohs L. and Smith S. D.** (2017) Bayesian estimation of the global biogeographical history of the Solanaceae. *Journal of Biogeography*, **44**, 887–899. https://doi.org/10.1111/jbi.12898

**FitzJohn R. G.** (2010) Quantitative Traits and Diversification. *Systematic Biology*, **59**, 619–633. https://doi.org/10.1093/sysbio/syq053

**FitzJohn R. G., Maddison W. P. and Otto S. P.** (2009) Estimating trait-dependent speciation and extinction rates from incompletely resolved phylogenies. *Systematic Biology*, **58**, 595–611. https://doi.org/10.1093/sysbio/syp067

**Rabosky D. L., Grundler M., Anderson C., Title P., Shi J. J., Brown J. W., Huang H. and Larson J. G.** (2014) BAMMtools: An R package for the analysis of evolutionary dynamics on phylogenetic trees. *Methods in Ecology and Evolution*, **5**, 701–707. https://doi.org/10.1111/2041-210X.12199
